# Bio-Activity and Dereplication-Based Discovery of Ophiobolins and Other Fungal Secondary Metabolites Targeting Leukemia Cells

**DOI:** 10.3390/molecules181214629

**Published:** 2013-11-26

**Authors:** Tanja Thorskov Bladt, Claudia Dürr, Peter Boldsen Knudsen, Sara Kildgaard, Jens Christian Frisvad, Charlotte Held Gotfredsen, Martina Seiffert, Thomas Ostenfeld Larsen

**Affiliations:** 1 Department of Systems Biology, Technical University of Denmark, Søltofts Plads, Building 221, Kgs. Lyngby DK-2800, Denmark; E-Mails: ttb@bio.dtu.dk (T.T.B.); pebok@bio.dtu.dk (P.B.K.); sarki@bio.dtu.dk (S.K.); jcf@bio.dtu.dk (J.C.F.); 2 German Cancer Research Center, Molecular Genetics, Im Neuenheimer Feld 280, Heidelberg D-69120, Germany; E-Mail: C.Duerr@dkfz-heidelberg.de; 3 Department of Chemistry, Technical University of Denmark, Kemitorvet, Building 201, Kgs. Lyngby DK-2800, Denmark; E-Mail: chg@kemi.dtu.dk

**Keywords:** natural products, ophiobolin U, dereplication, explorative solid phase extraction (E-SPE), filamentous fungi, cytotoxic, cancer, leukemia

## Abstract

The purpose of this study was to identify and characterize fungal natural products (NPs) with *in vitro* bioactivity towards leukemia cells. We based our screening on a combined analytical and bio-guided approach of LC-DAD-HRMS dereplication, explorative solid-phase extraction (E-SPE), and a co-culture platform of CLL and stromal cells. A total of 289 fungal extracts were screened and we tracked the activity to single compounds in seven of the most active extracts. The novel ophiobolin U was isolated together with the known ophiobolins C, H, K as well as 6-epiophiobolins G, K and N from three fungal strains in the *Aspergillus* section *Usti*. Ophiobolins A, B, C and K displayed bioactivity towards leukemia cells with induction of apoptosis at nanomolar concentrations. The remaining ophiobolins were mainly inactive or only slightly active at micromolar concentrations. Dereplication of those ophiobolin derivatives possessing different activity in combination with structural analysis allowed a correlation of the chemical structure and conformation with the extent of bioactivity, identifying the hydroxy group at C3 and an aldehyde at C21, as well as the A/B-*cis* ring structure, as indispensible for the strong activity of the ophiobolins. The known compounds penicillic acid, viridicatumtoxin, calbistrin A, brefeldin A, emestrin A, and neosolaniol monoacetate were identified from the extracts and also found generally cytotoxic.

## 1. Introduction

Screening and discovery of compounds that act against chronic lymphocytic leukemia (CLL) cells are crucial since CLL is considered as an incurable disease and currently applied treatment strategies primarily aim at prolonging patient survival [1,2]. Filamentous fungi have proven to be an incredible source of diverse bioactive compounds. The continuous improvements of analytical instruments and new approaches for fast dereplication have resulted in an increased interest in natural products discovery [3,4,5]. CLL is the most common type of leukemia among adults in the Western World. Even though most patients initially show a good response to therapy, relapse of disease is very frequent, with a subsequent increase in chemoresistance [6]. Consequently, there is a great need for discovery and development of new agents. Treatment strategies used today are based on small-molecule alkylating agents such as chlorambucil and fludarabine. These agents are often used in combination with monoclonal antibodies, or more recently inhibitors that target essential signaling pathways in CLL [7,8]. In contrast to other cancer cells, the majority of CLL cells is non-proliferating, arrested in G_0_/G_1_ phase of the cell cycle and accumulates in the patients due to apoptosis resistance [1]. *In vivo*, CLL cells are associated with a survival-inducing microenvironment of stromal cells, non-malignant leukocytes, so-called nurse-like cells, as well as growth and differentiation factors [9,10]. Removed from their natural microenvironment, the CLL cells rapidly undergo apoptosis *in vitro*, even though they are long-living cells *in vivo* [11]. However, viability of CLL cells can be maintained *in vitro* by co-cultivation with stromal cells, for example the bone marrow-derived cell line HS-5 [12,13]. Such co-cultures mimic the microenvironment of CLL cells *in vivo*, they are ideally suited for screening of natural products (NPs) [14]. We have already demonstrated that fungal NPs are a proper source for discovering compounds with activity towards CLL cells *in vitro*. This was done by identification of chaetoglobosin A from *P. aquamarinum*. Chaetoglobosin A induced apoptosis in CLL cells more selectively compared to healthy cells with a median lethal dose (LC_50_) of 2.8 µM [15].

Novel secondary metabolites produced by filamentous fungi such as *Penicillium* and *Aspergillus* are being discovered continuously [4,16]. With the increase in target-based specific biological assays, previously described compounds might display novel bioactivities [17,18], justifying their presence in novel screening efforts. Testing all known as well as novel NPs against all disease targets is an impracticable approach, why setting up a suitable strategy is essential to any screening program [19]. Targeted screening strategies in NPs-based drug discovery rely on identifying compounds targeting a specific disease or biological mechanism. In such screening campaigns, the selection of fungi is essential and needs to represent as wide a biodiversity as possible, with the hope of an equally high chemodiversity [19]. One strategy to increase chemodiversity for rapid bio-testing is to select strains representing a wide variety of species with a limited number of strains from each species [19,20]. The spectrum of compounds produced by the individual strains can further be diversified or maximized by the ‘one strain-many compounds’ (OSMAC) approach, through variation of culture conditions [16].

Dereplication is the tentative identification of known NPs in complex mixtures, before unnecessary time is spent on isolating already known compounds. One dereplication approach is based on liquid chromatography-diode array detection-high resolution mass spectrometry (LC-DAD-HRMS) and database searching, which ensures a high throughput and reproducibility [3]. Subsequent to dereplication a separation strategy for preparative isolation of NPs is necessary. Here a small scale preliminary chemical characterization focusing on identification of functional groups is helpful. Co-eluting interferences often experienced in traditional reverse phase (RP) chromatography can be reduced or even completely removed by choosing orthogonal purifications strategies [21]. One approach for prefractionation is explorative solid-phase-extraction (E-SPE) [21] that relies on ion-exchanger columns such as strong anion-exchanger (SAX), mixed mode anion-exchanger (MAX), and strong cation-exchanger (SCX). This method has proven to be very powerful for separating fungal NPs due to the relative high percentages of ionizable functional groups [21]. 

In this current paper we describe our screening efforts of discovering fungal NPs with bio-activity in a co-culture platform of chronic lymphocytic leukemia (CLL) cells and stromal cells and their retesting in CLL cells cultures in conditioned media of stromal cells [12]. The fungal NPs were tentatively identified by LC-DAD-HRMS based dereplication and extracts were fractionated in order to assign the activity to single compounds [3]. Confidence in this identification was improved by using an E-SPE strategy based on an array of orthogonal separation techniques [19,21]. Thereby among others, ophiobolins were identified and further structural studies were performed by dereplicating different ophiobolin dervatives leading to the identification of chemical moieties and conformations that are indispensable for strong bioactivity. Ophiobolins lacking these moieties possessed either low or no bioactivity.

## 2. Results and Discussion

**Figure 1:**
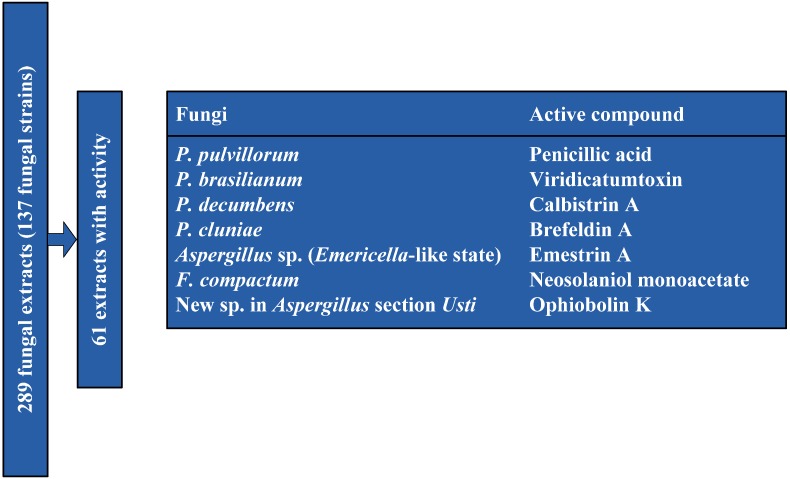
Screening set-up. Two hundred and eighty nine (289) fungal extracts (from 137 fungal strains) were tested for cell death-inducing activity for CLL cells but not for stromal cells. From the 61 active extracts the seven candidates that displayed the highest bio-assay activity were selected for single compound isolation in a large scale and further bio testing.

A total of 289 fungal extracts were prepared from cultivation of 137 fungal strains (Figure 1 and Table S1) on a selection of solid media at variable temperatures in accordance with the OSMAC approach [16]. The extracts were prepared by the micro-extraction method developed by Smedsgaard [22]. To obtain a representative sample of the fungal colonies the plugs were taken across the colony. Sixty one (61) extracts showed activity towards CLL cells (Table S2). Seven of the candidates that displayed the highest level of activity (Figure 1) were selected for further bio-testing. Large-scale extracts were prepared from incubation on the media supporting the highest level of bioactivity, and the extracts were prefractionated before further testing.

### 2.1. MS Based Dereplication of Penicillium pulvillorum Extract

**Figure 2:**
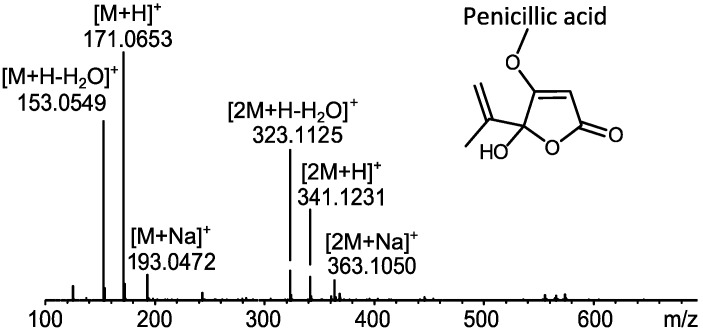
Dereplication of penicillic acid from *P. pulvillorum* mass spectrum of penicillic acid [23]. The mass spectrum shows a widespread adduct pattern that besides [M+H]^+^ contains ions that corresponds to neutral loss of water [M+H-H_2_O]^+^ and the sodiated adduct [M+Na]^+^, as well as the corresponding dimeric ions [2M+H]^+^, [2M+H-H_2_O]^+^, and [2M+Na]^+^.

The extract of *P. pulvillorum* (IBT 22393) was among the candidates that displayed the highest level of activity (≈1.25 µg/mL). The first five flash fractions (ranging from 15%–40% organic) were tested active against CLL cells *in vitro*. LC-DAD-HRMS revealed one major component shared between these fractions, with an elementary composition of C_8_H_10_O_4_ (−0.7 ppm mass accuracy) (Figure 2). AntiBase2012 [23] revealed penicillic acid as a likely candidate responsible for the observed activity. The tentative identification of penicillic acid was confirmed by comparison of the retention time to a standard from our in-house metabolite database (1,559 standards) as well as comparison of ^1^H- and ^13^C-NMR chemical shifts to the literature data [24].

To verify the observed anti-leukemic activity in the extract, penicillic acid was purified and tested on CLL cells, resulting in induced cell death in both CLL and stromal (HS-5) cells. No further work on this extract was done as penicillic acid is regarded as a generally cytotoxic compound [24].

### 2.2. Comparative Dereplication Based on Explorative Solid Phase Extraction (E-SPE)

**Figure 3:**
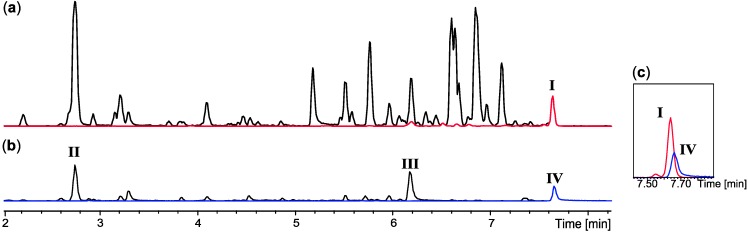
E-SPE strategy based on a SAX column to separate co-eluting compounds in the crude extract of *P. brasilianum*. (**a**) UHPLC chromatograms of the SAX fraction that contained neutral/basic compounds. The ion trace of compound I (*m/z* 462.2387) is marked with red; (**b**) UHPLC chromatogram of the SAX fraction that contained acidic compounds. The ion trace of compound IV (*m/z* 548.1916) is marked with blue; (**c**) In the crude extract compound I and IV were co-eluting on a RP C_18_ column.

The E-SPE strategy was applied to a series of highly complex extracts, *i.e.*, *P. brasilianum* (IBT 22244), *P. decumbens* (IBT 11843), *P. cluniae* (IBT 21051), *Aspergillus* sp. (*Emericella*-like state) (IBT 22838), and *Fusarium compactum* (IBT 9034). The extract of *P. brasilianum* (IBT 22244) was very potent against CLL cells *in vitro* (≈5 ng/mL) with the active compound retained on both anion-exchangers (SAX and MAX) as well as the two normal-phase columns (diol and amino), while unretained on the cation-exchanger (SCX). The combined biological and chromatographic information lead to the conclusion that the bioactive compound contained a strong anion. The large scale extract was fractionated on a SAX column. Comparison of chromatographic peaks from the fraction that contained neutral/basic compounds (Figure 3a) and the fraction with acidic compounds (Figure 3b), showed that the anion-exchange was extremely selective, removing the majority of inactive compounds from the extract.

**Figure 4:**
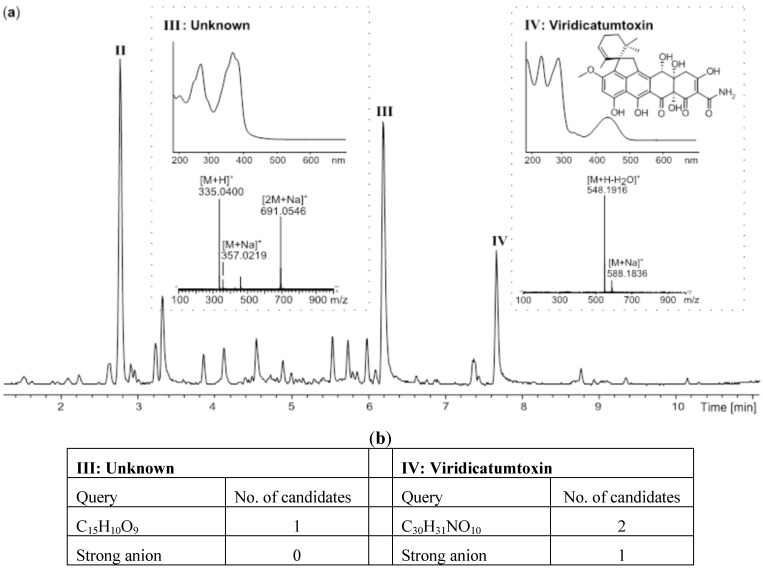
Dereplication of the *P. brasilianum extract* (**a**) UHPLC chromatogram of the active SAX fraction that contained the acidic compounds as well as UV and MS spectra of the potential candidates; (**b**) Hits in Antibase2012.

In the initial extract, the two compounds **I** and **IV** co-eluted on a C_18_ RP column (Figure 3c). These were easily and quantitatively separated on the SAX column due to the difference in charged functionalities. Only three major acidic compounds were left in the bioactive fraction (Figure 3b and Figure 4a), significantly simplifying the subsequent dereplication and purification process. Based on comparative HRMS analysis, compound **II** was immediately eliminated due to its presence in the inactive neutral/basic fraction. The molecular formula of compounds **III** and **IV** were established as C_15_H_10_O_9_ (−0.7 ppm) and C_30_H_31_NO_10_ (−0.2 ppm), respectively. These were used as queries in AntiBase2012 (Figure 4b) [23]. Compound **II** had no hits in AntiBase2012 that contained a strong anion, thus likely being a novel compound or novel analogue of a known compound. Compound **IV** had two hits in AntiBase2012. One of the candidates had no strong anion and was consequently eliminated, which left viridicatumtoxin as the only candidate (Figure 4b).

The identity of viridicatumtoxin as compound **IV** was confirmed: (1) by comparison to the retention time and UV spectrum of an in-house standard; (2) by the fact that it held a strong anion; and (3) by having similar ^1^H-NMR chemical shifts as published for viridicatumtoxin [25]. Viridicatumtoxin was isolated as one of the most cytotoxic compounds tested towards CLL cells in this screening campaign with a median lethal concentrations (LC_50_) value between 0.7 and 3.5 nM. Further testing revealed that the activity was not specific, as both CLL and stromal cells were targeted.

**Figure 5:**
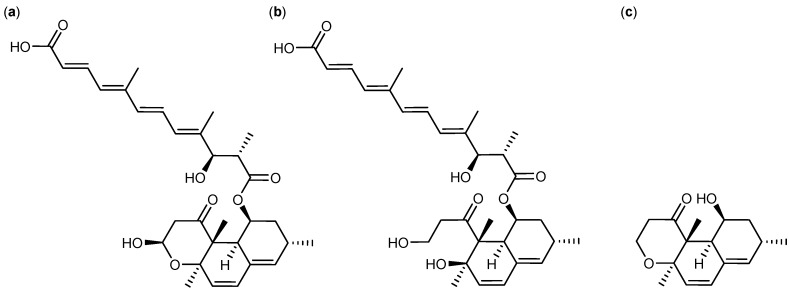
Structure of (**a**) Calbistrin A, (**b**) Calbistrin C, and (**c**) Versiol.

One flash fraction (70% organic) from the *P. decumbens* (IBT 11843) extract was found active towards CLL cells (≈200 ng/mL). Further E-SPE analysis showed that the active compound was retained on SAX and MAX columns indicating the presence of a strong anion. By comparative dereplication tentative identifications of calbistrin A (Figure 5a) and B as well as their precursor (or decomposition product) versiol (Figure 5c) were established within the fraction. The MS based dereplication was complicated by the fact that the [M+H]^+^ ion was absent in the mass spectra of calbistrin A and B. The presences of strong adduct- and fragmentation patterns consisting of the sodiated, [M+Na]^+^, and the ammoniated, [M+NH_4_]^+^, adducts as well as neutral loss of one and two water molecules assisted the establishment of the monoisotopic masses and hereby the molecular formulas of calbistrin A and B. The identity of calbistrin A was confirmed by the presence of a carboxylic acid and by comparison of retention time and UV spectrum to an in-house standard. The tentative identity of calbistrin B was confirmed by comparison of the UV spectrum to that of calbistrin A (Figure 5a). Testing of calbistrin A from our in-house metabolite collection showed general cytotoxic activity towards CLL and healthy cells. Comparative experiments with calbistrin C (Figure 5b) from the metabolite collection did not induce cell death, indicating that the pharmacophore is located in the versiol part (Figure 5c) of the molecule.

**Figure 6:**
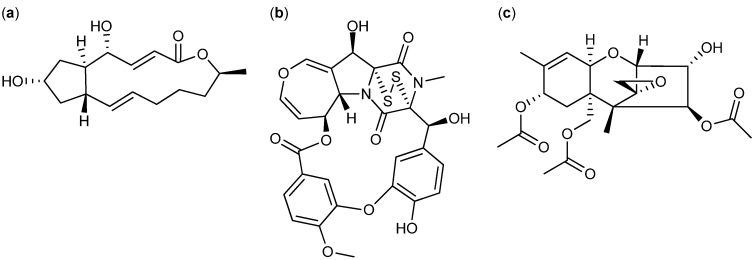
Examples of E-SPE and comparative dereplication (**a**) Brefeldin produced by *P. cluniae*, (**b**) Emestrin A produced by *Aspergillus* sp. (*Emericella*-like state), and (**c**) Neosolaniol monoacetate produced by *F. compactum*.

A bioactive flash fraction (activity approx. 100 ng/mL) from a *Penicillium cluniae* (IBT 21051) extract was likewise subjected to E-SPE. Here, the bioactivity profiled revealed that the active compound was a medium to apolar compound with no charged functionalities. By comparative dereplication, the active compound was tentatively identified as brefeldin A (Figure 6a), which was in accordance with the profile revealed by E-SPE. The identity of brefeldin A was confirmed by its retention time and UV spectrum compared to an in-house standard. Brefeldin A is a known anticancer compound [5,26] and commercially available, thus the activity was easily confirmed in the CLL assay. The compound displayed general cytotoxic activity for CLL cells (0.39–1.56 µM) and stromal cells.

The E-SPE strategy of the bioactive extract (≈40 ng/mL) from *Aspergillus* sp. (*Emericella*-like state, IBT 22838) resulted in retention of the bioactive compound on the amino normal phase SPE column. Fast comparative dereplication based on UV spectra and retention times of in-house standards as well as comparison of ^1^H-NMR chemical shifts [27] led to an identification of the known antifungal and anticancer compound, emestrin A (Figure 6b) [5,28,29]. The pure emestrin A isolated form the active fractions showed cytotoxic activity towards CLL cells and stromal cells at the same concentration levels. Accordingly, emestrin A is regarded as a generally cytotoxic compound with no therapeutic window [30]. No further work was pursued on the *Aspergillus* sp. (*Emericella*-like state) extract.

The last example of bio-guided isolation based on E-SPE is demonstrated by the *F. compactum* (IBT 9034) extract with an activity at approximately 200 ng/mL. The bioactive compound from *F. compactum* was retained on both the diol and amino columns in the E-SPE pre-fractionation experiment. Comparative dereplication revealed only one candidate that might be responsible for the activity. The compound was tentatively identified as the known trichothecene, neosolaniol monoacetate (Figure 6c). The compound was isolated and the ^1^H-NMR data was compared to the literature for final identification of neosolaniol monoacetate [31]. Neosolaniol monoacetate was tested in the CLL assay and found as a generally cytotoxic why no further work was performed on the *F. compactum* extract. The E-SPE approach with the optimized collection of ion-exchangers and normal phase SPE columns has turned out to be a good combination to evaluate and follow bioactivity of fungal extracts.

### 2.3. Biological Structure-Activity Relationship of Ophiobolins

**Figure 7:**
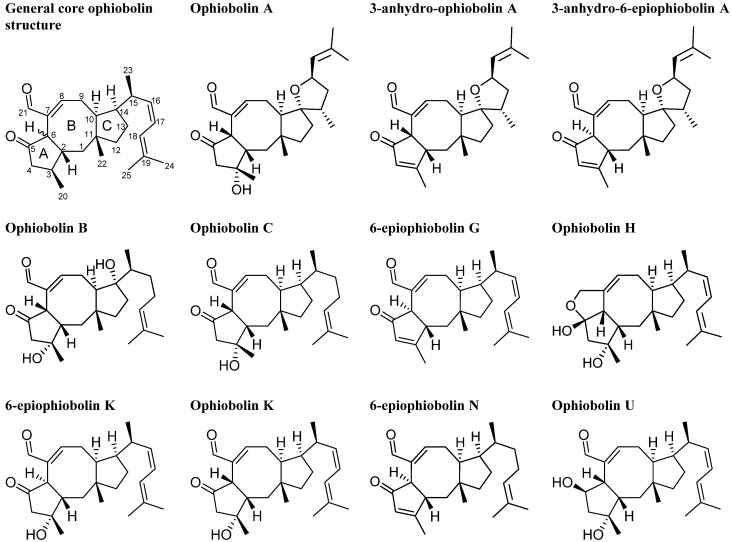
Structures of ophiobolin A, 3-anhydro-ophiobolin A, 3-anhydro-6-epiophiobolin A, ophiobolin B, ophiobolin C, 6-epiophiobolin G, ophiobolin H, 6-epiophiobolin K, ophiobolin K, 6-epiophiobolin N, and ophiobolin U.

The bioactive extract from a new species in *Aspergillus* section *Usti* (IBT 18591) was more selective than the above mentioned active extracts and in consequence selected for more detailed investigations. MS- and UV-based dereplication led to the tentative identification of the ophiobolin family of compounds. Ophiobolin K and 6-epiophiobolin K (Figure 7) [32] were isolated and ophiobolin K was found very potent against CLL cells *in vitro*.

The ophiobolins are a family of naturally occurring sesterterpenoids, currently comprising more than 35 known analogues [33,34,35,36,37]. They all consist of a C_25_ skeleton with a dicyclopenta[a,d]cyclooctane ring system. Some ophiobolins have an extra ring incorporated, as observed in ophiobolin A and H (Figure 7), forming two different types of tetra-cyclic structures [38]. The absolute configuration of ophiobolin A and G have been determined by X-ray crystallography [39,40] and the conformations of all stereocenters except C6 is expected to be conserved based on the biosynthetic production of ophiobolins demonstrated by the first sesterterpene synthase described in 2013 [41]. Ophiobolins exhibit a broad spectrum of inhibitory activity against cancer cell lines, including lung cancer A549, breast cancer MCF7, colon cancer HT29, melanoma Mel20, leukemia P388 and L1210 cell lines [5,34,35,42,43,44,45]. 

**Figure 8:**
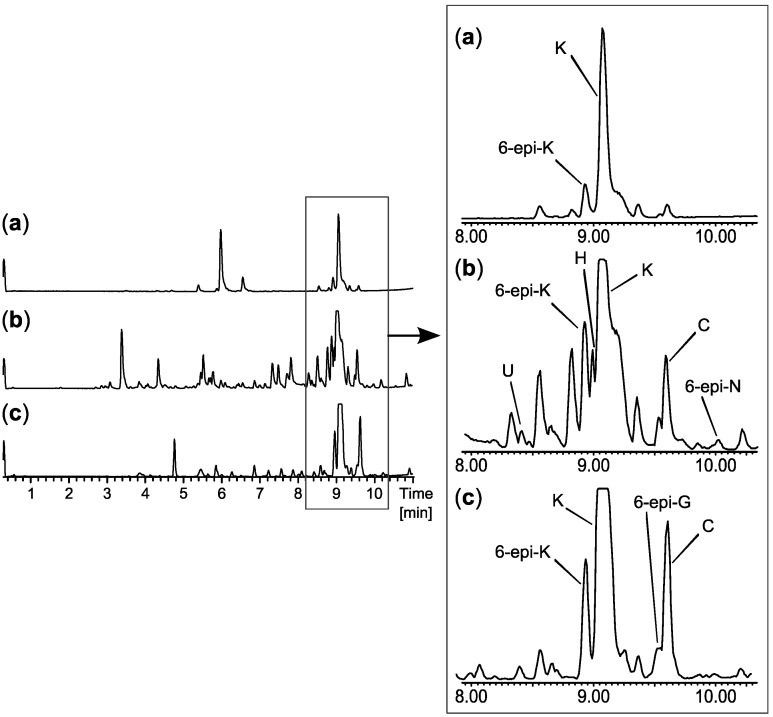
UHPLC chromatograms of (**a**) the new species in *Aspergillus* section *Usti* (IBT 18591) producing ophiobolin K and 6-epiophiobolin K, (**b**) *A. insuetus* (IBT 28266) producing the novel ophiobolin U together with ophiobolin H, K, C as well as 6-epiophiobolin K and N and (**c**) *A. calidoustus* (IBT 25726) producing ophiobolin K and C as well as 6-epiophiobolin K and G.

Further investigations of the anti-leukemic activity and pharmacophore of the ophiobolins in the CLL/stromal cell co-culture platform were performed with the purpose of isolating a high number of naturally occurring analogs as well as identification of novel analogues. Taking advantage of the huge biodiversity available in the IBT culture collection [19], we expanded the biodiversity and hereby the expected chemodiversity with 12 closely related *Aspergilli* from the section *Usti* (Table S7) [46]. Cultures of the 12 new strains were extracted in micro-scale [22] to explore their potential for producing ophiobolins. *A. insuetus* (IBT 28266) and *A. calidoustus* (IBT 25726) were identified as potent ophiobolin producers with one likely novel and more known ophiobolins analogs compared to the original strain (Figure 8).

**Figure 9:**
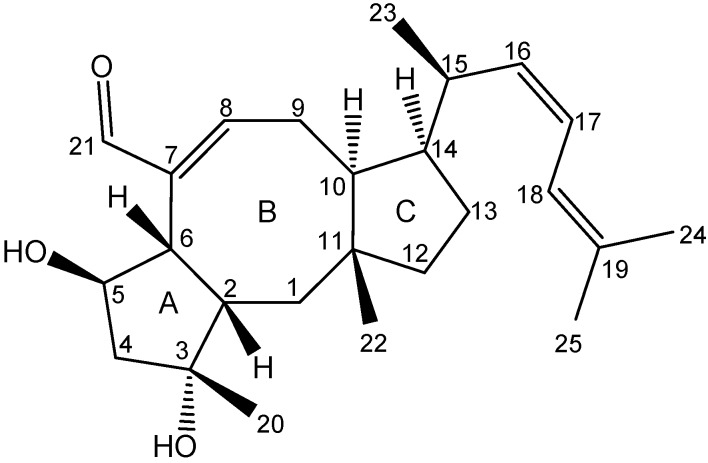
Structure of ophiobolin U.

The novel ophiobolin U (Figure 9) was isolated together with ophiobolin H [40] (Figure 7) and the rare 6-epiophiobolin N [43] (Figure 7) from the *A. insuetus* extract, while ophiobolin C [47] (Figure 7) and 6-epiophiobolin G [43] (Figure 7) were isolated from the *A. calidoustus* extract.

**Figure 10:**
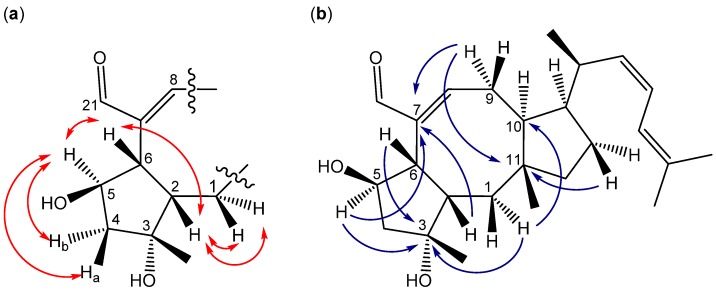
(**a**) Important DQF-COSY couplings and (**b**) important HMBC connectivities in the novel compound ophiobolin U.

The structure of the novel ophiobolin U was elucidated by 1D and 2D NMR spectroscopy. The ^1^H-NMR spectrum of ophiobolin U was closely related to that of ophiobolin K with many practically identical chemical shifts (Table S8 and S9). The most remarkable difference between ophiobolin U and ophiobolin K was found at C5 that shifted 143.9 ppm upfield from 217.0 to 73.1 ppm in the carbon spectrum, indicating the disappearance of a ketone group. C5 had an additional HSQC correlation to a signal at 4.91 ppm (H5). This significant change indicated a reduction of the ketone (C5) in ophiobolin K to a secondary alcohol in ophiobolin U. This reduction was confirmed by the identification of a COSY spin system between H1-H2-H6 in ophiobolin K that in ophiobolin U was expanded with a vicinal coupling between the protons at 3.02 (H6) and 4.91 (H5) and further a vicinal coupling between H5 and the diastereotopic protons at 1.87 (H4a) and 2.68 ppm(H4b) (Figure 10a).

The spin systems identified in the DQF-COSY spectrum of ophiobolin U were assembled through HMBC correlations, which also enabled the identification of the quaternary carbon atoms. The most important HMBC correlations are shown in Figure 10b. The COSY spin systems were connected by HMBC connectivities further confirming the presence of the eight-membered ring. HMBC connectivities were found from H5 to the quaternary carbons at 81.9 (C3) and 142.1 ppm (C7), from the diastereotopic protons at 1.03 (H1a) and 1.58 ppm (H1b) to C3 and the carbon at 54.0 ppm (C10), and finally from H9 to C7 and the quaternary carbon at 44.1 ppm (C11). The reduction at C5 changed the chemical environment of the surrounding carbons (C2, C3, C6, C8 and C21) that were more deshielded and therefore shifted 1.8–6.5 ppm downfield compared to ophiobolin K (Table S8). The remaining chemical shifts in ophiobolin U matched the chemical shifts of ophiobolin K (Tables S8 and S9).

**Figure 11:**
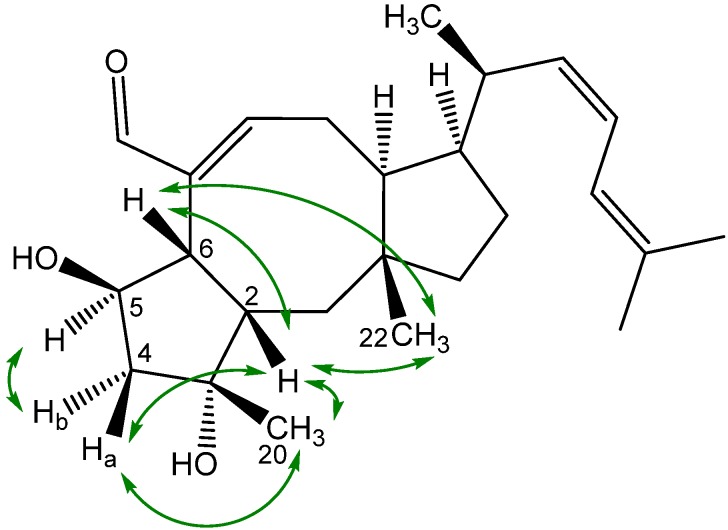
Important NOE correlations in ophiobolin U.

The stereochemistry of the A/B ring system in ophiobolin U (Figure 9) was assigned based on NOE correlations and chemical shifts. The A/B-*cis* ring system was established by the NOE correlations found between the protons at 2.30 (H2) and 3.02 ppm (H6), as demonstrated in Figure 11. The stereochemistry of C-5 was tentatively assigned through strong NOE correlations of the diastereotopic protons at 1.87 (H4a) and 2.68 ppm (H4b). H4a had NOE correlations to the protons at 1.26 ppm (H20) and H2, while H4b had a NOE correlation to H5, which indicated that the hydroxy group at C5 was *cis* to H6. Other important NOE correlations are shown in Figure 11.

The A/B-*cis* was confirmed by chemical shifts. Earlier reports showed that C1 and C22 in ophiobolins with A/B-*cis* ring structure are more upfield compared to ophiobolins with A/B-*trans* ring structure [48]. In ophiobolin U and ophiobolin K, the chemical shifts of C1 and C22 were more upfield compared to 6-epiophiobolin K, which indicate a A/B-*cis* ring structure in ophiobolin U. Other reports have showed that the A/B-*cis* ring structure in ophiobolins causes a small deshielding (0.2–0.3 ppm) for H2 and H8 as well as a small shielding (0.3–0.6 ppm) for H4 [49]. The ^1^H chemical shifts for ophiobolin U were inconclusive regarding conformation of the A/B ring system. The residual stereocenters of ophiobolin U were the same as in the known ophiobolins due to the stereospecificity of the biosynthetic pathway of the ophiobolins [41,50].

The stereochemistry of the A/B ring system of the remaining six ophiobolins (ophiobolin K, 6-epiophiobolin K, 6-epiophiobolin N, 6-epiophiobolin G, ophiobolin H, and ophiobolin C) isolated in this study were confirmed by NOE correlations. Together with the general trend that C1 and C22 in the ophiobolins with A/B-*cis* ring structure were more upfield compared to ophiobolins with A/B-*trans* ring structure [43,48]. The shifting of chemical shifts for H2, H4, and H8 for the A/B-*cis* ring system were more ambiguous due to the small deshielding/shielding in chemical shifts and inconclusive for the seven ophiobolins. A comparison of the ^13^C and ^1^H chemical shifts of all the seven ophiobolins are found in Tables S8 and S9, respectively.

**Figure 12:**
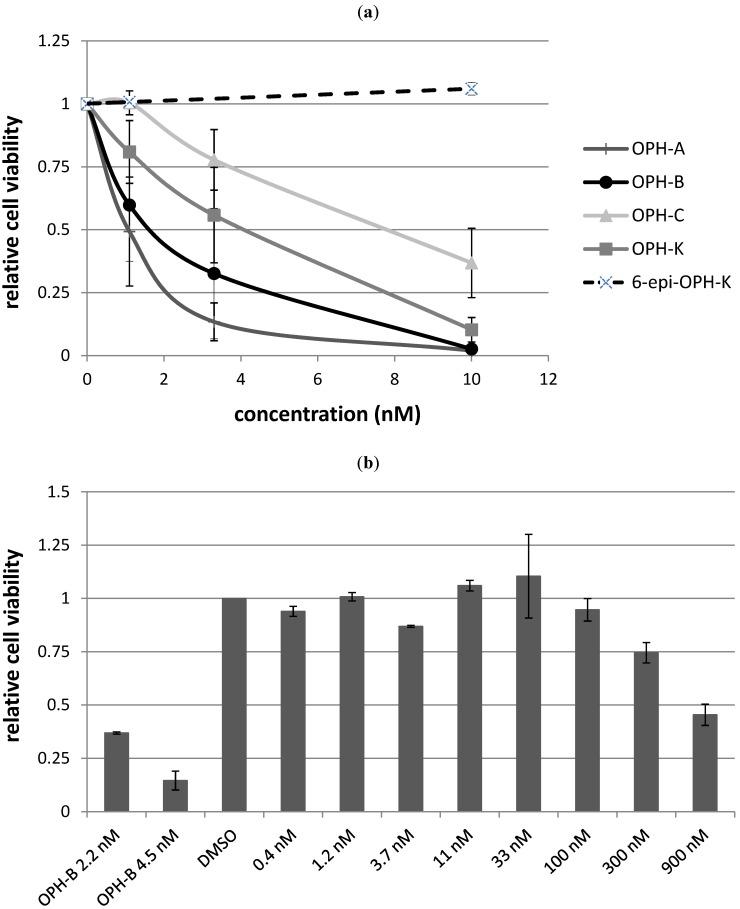
Effects of different ophiobolins on CLL cell viability. (**a**) CLL cells cultured in HS-5 conditioned media were treated for 24 h with increasing concentrations of ophiobolin A, ophiobolin B, ophiobolin C, ophiobolin K, and 6-epiophiobolin K, and cell viability was analyzed by CellTiter-Glo^®^ assay measuring each data point as duplicate. Relative cell viability compared to DMSO control (0.1%) is depicted as mean values +SD of 3 independentCLL samples; (**b**) As 6-epiophiobolin K treatment did not decrease cell viability in the concentration range tested in (a), CLL cells were treated with up to 900 nM of 6-epiophiobolin K, and cell viability was determined and compared to the active ophiobolin B.

Besides the seven purified ophiobolins: ophiobolin A, ophiobolin B, 3-anhydroophiobolin A, and 3-anhydro-6-epiophiobolin A (Figure 7) were bought as standards with the aim of obtaining a broader understanding of the SAR of the ophiobolin family against CLL cells. Ophiobolin U was unstable and therefore not applied in any bioassay, but the remaining ten ophiobolins were tested for their cytotoxic activity towards CLL cells. Ophiobolin A, B, C, and K showed the strongest effects with LC_50_ values between 1 and 8 nM (results compiled in Table 1 and Figure 12a). Testing of normal lung fibroblasts revealed that ophiobolin A and B displayed cytotoxic effects at 10 nM concentration indicating a slight difference in bio-activity for CLL cells in comparison to healthy fibroblasts. Ophiobolin C and K displayed no effect towards normal lung fibroblasts in concentrations up to 10 nM (Figure S17). Interestingly, 3-anhydroophiobolin A, 3-anhydro-6-epiophiobolin A, 6-epiophiobolin G, ophiobolin H, 6-epiophiobolin K, and 6-epiophiobolin N exhibited low or no activity towards CLL cells. In fact, 6-epiophiobolin K targeted CLL cell viability only when applied at a 100-fold higher concentration than ophiobolin K (Figure 12b). Ophiobolin-treated cells were further stained with PE-labelled Annexin-V and 7-AAD, or antibodies for activated caspase-3 prior to flow cytometric analyses. Thereby, apoptosis was identified as the mode of killing of CLL cells as demonstrated recently for chaetoglobosin A [15].

**Table 1:** Apoptosis inducing activity (LC_50_ [nM]) of the 10 ophiobolins towards CLL cells.

Compound	LC_50_
Ophiobolin A	1 nM
3-anhydro-ophiobolin A	Inactive
3-anhydro-6-epiophiobolin A	Inactive
Ophiobolin B	2 nM
Ophiobolin C	8 nM
6-epiophiobolin G	Inactive
Ophiobolin H	Inactive
Ophiobolin K	4 nM
6-epiophiobolin K	Inactive
6-epiophiobolin H	Inactive

**Figure 13:**
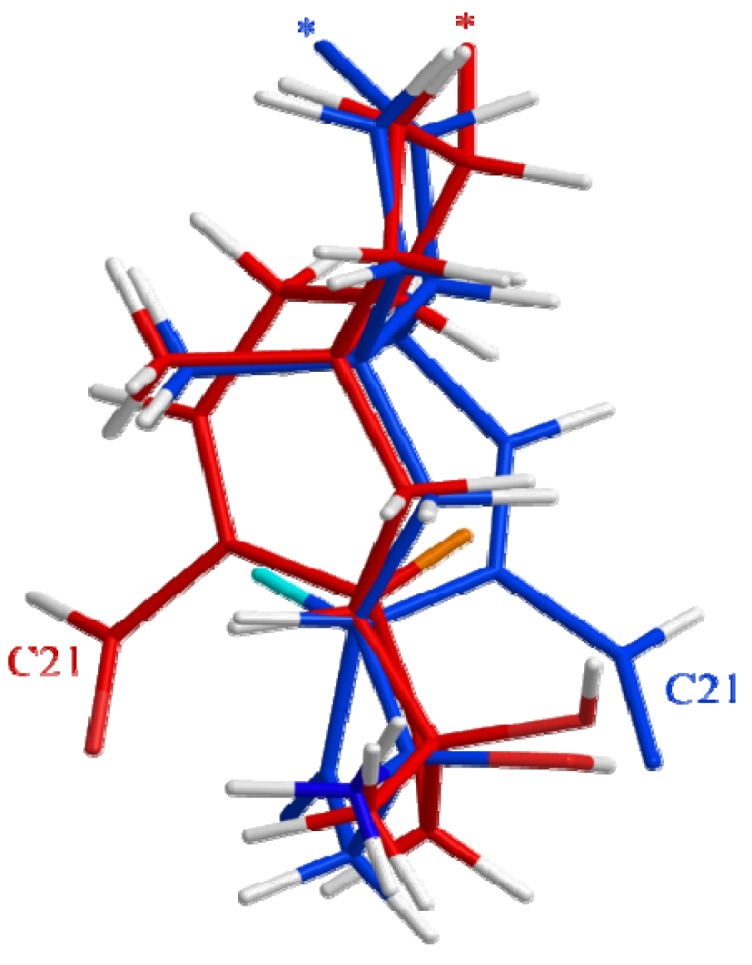
Modulated 3D structures of ophiobolin K (blue) and 6-epiophiobolin K (red) in their lowest energy conformations overlaid. H6 protons are marked in ophiobolin K (cyan) and 6-epiophiobolin K (orange). The chain extending from C14 in ring C is not displayed to get a better clarity of the structures (the cut-off point is marked with *).

The results displayed in Table 1 and Figure 12a indicates that presence of a hydroxy group at C3 and an aldehyde at C21 is crucial for the activity of the ophiobolins. Our findings are thus in agreement with previous studies that have shown that these two groups covalently bind to calmodulin [51,52]. None of the 6-epiophiobolins tested were active against the CLL cells. To earn a broader understanding of the importance of this small steric change at C6, 3D-modeling of ophiobolin K (blue) and 6-epiophiobolin K (red) were done to give a visual representation of their lowest energy conformations (Figure 13).

The difference in conformation between ophiobolin K and 6-epiophiobolin K involves a flipping of the eight-membered ring that result in a change of position of the C21 aldehyde from one to the other side of the plane observed in the figure. This conformational change is likely preventing the binding of calmodulin by the C21 aldehyde due to steric hindrance, resulting in the lack of activity for 6-epiophiobolin K contrasting ophiobolin K. 

## 3. Experimental

### 3.1. General

The fungal strains used are from the IBT culture collection at Department of Systems Biology, Technical University of Denmark. The LC-MS analyses were performed on a maXis quadrupole time of flight (qTOF) mass spectrometer (Bruker Daltonics, Bremen, Germany) with an electrospray ionization (ESI) ion source. The maXis was calibrated using sodium formate automatically infused prior to each analytical run, providing a mass accuracy of below 1 ppm. The mass spectrometer was linked to an Ultimate 3000 UHPLC system (Dionex, Sunnyvale, CA, USA) with DAD. Separation was achieved on a Kinetex C_18_, 2.6 µm, 2.1 × 100 mm column (Phenomenex, Torrance, CA, USA) with a flow of 0.4 mL min^−1^ at 40 °C using a linear gradient 10% acetonitrile (ACN) in Milli-Q water (MQ) with 20 µM formic acid (FA) going to 100% ACN in 10 min. All compounds were isolated by bio-guided fractionation started by flash chromatography of the crude extracts, fractionated with an Isolera One automated flash system (Biotage, Uppsala, Sweden). The isolation of compounds were performed by a semi-preparative Gilson HPLC system (Middleton, WI, USA) with a 215 Liquid Handler, 819 Injection Module and a 172 DAD and fully controlled with Trilution LC software or on a Waters 600 chromatograph (Milford, MA, USA) attached to a Waters 600 DAD. One-dimensional and two-dimensional NMR experiments were acquired using standard pulse sequences on a 800 MHz Bruker Avance spectrometer with a 5 mm TCI cryoprobe at the Danish Instrument Centre for NMR Spectroscopy of Biological Macromolecules at Carlsberg Laboratory, alternatively on a 500 MHz Varian Unity Inova (Palo Alto, CA, USA) equipped with a HCP probe or a 400 MHz Bruker Avance III equipped a BBO Prodigy cryoprobe NMR spectrometers.

### 3.2. Micro Extraction for Initial Screen

Two hundred and eighty nine (289) fungal extracts were prepared from cultivation of 137 fungal strains (Table S1). All extracts were prepared in accordance with the micro-extraction method developed by Smedsgaard [22]. Five plugs were collected across the colony. The samples were subsequently extracted using (3:2:1 v/v/v) methanol (MeOH), dichloromethane (DCM) and ethyl acetate (EtOAc) with 0.5% FA.

### 3.3. Cultivation and Extraction

The seven extracts described here were *P. pulvillorum* (IBT 22393), *P. brasilianum* (IBT 22244), *P. decumbens* (IBT 11843), *P. cluniae* (IBT 21051), *Aspergillus* sp. (*Emericella*-like state, IBT 22838), *F. compactum* (IBT 9034), and a new species in *Aspergillus* section *Usti* (IBT 18591). Each fungus was cultivated on 50 plates (media are listed in supplementary) for 8 days at 25 °C in the dark with the exceptions of *P. brasilianum*. *P. brasilianum* (IBT 22244), *A. insuetus* (IBT 28266), and *A. calidoustus* (IBT 25726) were cultivated on 200 agar plates with Yeast Extract Sucrose (YES) for 14 days (except *A. calidoustus* that was incubated for 7 days) at 25 °C in the dark. All the fungi were extracted separately with EtOAc containing 1% FA. Unwanted carbohydrates from the media as well as fatty acids were removed from the four large extract (*P. brasilianum*, the new species in *Aspergillus* section *Usti*, *A. insuetus*, and *A. calidoustus*) by liquid-liquid extraction with water/MeOH and heptane, respectively leaving the crude extracts.

### 3.4. Bioassay-Guided Fractionation

The crude extract of *P. decumbens*, *P. pulvillorum*, *P. cluniae, Aspergillus* sp. (*Emericella*-like state), and *F. compactum* were fractionated on a RP C_18_ (25 g, 33 mL) column flash column with at gradient of: 15%–100% ACN in 20 min and flow rate 25 mL/min. No further fractionations were done with the bioactive flash fraction from the *P. cluniae* extract. Penicillic acid was purified from the bioactive flash fraction from the *P. pulvillorum* extract by semi-preparative HPLC LunaII C_18_ (250 × 10 mm, 5 µm) column with at gradient of: 15%–100% ACN in 20 min and flow rate 5 mL/min. ACN and MQ were added 20 mM FA.

*Penicillic acid*: White solid; UV (ACN) λ_max_: 227 nm; HRMS *m/z* 170.0573 (M^+^ calculated for C_8_H_10_O_4_, *m/z* 170.0574; 0.5 ppm).

#### 3.4.1. E-SPE

*P. brasilianum*, *P. decumbens*, *Aspergillus* sp. (*Emericella*-like state), and *F. compactum* extracts were prefractionated analytical in accordance with the E-SPE method developed by Månsson *et al.* [21] with SAX, MAX, and SCX ion-exchanger SPE columns though without the Sephadex LH-20 column. Additionally two normal phase (amino and diol) columns were add to the setup. For each extract, 5 mg was loaded on both the amino column (100 mg, 1 mL) and the diol column (100 mg, 1 mL). The compounds were eluted by 2 column volume (CV) heptane, 2 CV DCM, 2 CV DCM/EtOAc (1:1), 2 CV EtOAc, 2 CV EtOAc/MeOH, 2 CV MeOH, 4 CV ACN, 2 CV ACN/MQ + 2% FA (75:25), 2 CV ACN/MQ + 2% FA (50:50), 2 CV ACN/MQ + 2% FA (25:75), 2 CV ACN/MQ + 2% FA (10:90). No further fractionations were done with the bioactive flash fraction from the *P. decumbens* extract.

The bioactive C_18_ flash fraction from the *Aspergillus* sp. (*Emericella*-like state) extract was loaded on the amino column (10 g, 15 mL) and eluted by 2 CV heptane, 2 CV DCM, 2 CV DCM/EtOAc (1:1), 2 CV EtOAc, 2 CV EtOAc/MeOH, 2 CV MeOH, 4 CV ACN, 2 CV ACN/MQ + 2% FA(75:25), 2 CV ACN/MQ + 2% FA (50:50), 2 CV ACN/MQ + 2% FA (25:75), 2 CV ACN/MQ + 2% FA (10:90). Emestrin A (1.1 mg) was finally isolated by preparative HPLC on a LunaII C_18_ (250 × 10 mm, 5 µm) column with at gradient of: 50%–100% ACN in 20 min and flow rate 5 mL/min. ACN and MQ were added 50 ppm trifluoroacetic acid (TFA).

*Emestrin A*: White solid; [α]_589.3nm_: +22°; UV (ACN) λ_max_: 230 (sh), 267 (sh), 285 (sh); HRMS *m/z* 598.0713 (M^+^ calculated for C_27_H_22_N_2_O_10_S_2_, *m/z* 598.0711; 0.4 ppm). 

The bioactive C_18_ flash fraction from the *F. compactum* extract was loaded on a diol column (10 g, 15 mL) and eluted by 2 CV heptane, 2 CV heptanes/DCM (1:1), 2 CV DCM, 2 CV DCM/EtOAc (1:1), 2 CV EtoAc, 2 CV EtOAc/MeOH, 2 CV MeOH, 2 CV MeOH/ACN (1:1) and 2 CV ACN. Neosolaniol monoacetate was isolated with semi-preparative HPLC on a Luna II, C_18_, 5 µm, 10 × 250 mm column with 30%–70% ACN in 20 min and flow rate 5 mL/min. ACN and MQ were added 20 mM FA.

*Neosolaniol monoacetate*: White solid; HRMS *m/z* 424.1728 (M^+^ calculated for C_21_H_28_O_9_, *m/z* 424.1728; 0 ppm).

The crude extract of *P. brasilianum* was loaded on a SAX column (100 g, 132 mL) and washed by 1 CV 70% MeOH in MQ (pH 11) and 1 CV 100% MeOH (pH 7) giving the SAX-1 fraction. Subsequently eluted by 2 CV 100% MeOH (pH 2) giving the SAX-2 fraction. Viridicatumtoxin was isolated (27.5 mg) from SAX-2 on a RP C_18_ column with at gradient of: 30%–100% ACN in 60 min and flow rate 40 mL/min. ACN and MQ were added 50 ppm TFA.

*Viridicatumtoxin*: Yellow solid; UV (ACN) λ_max_: 238, 286, 435 nm; HRMS *m/z* 565.1942 (M^+^ calculated for C_30_H_31_NO_10_, *m/z* 565.1942; 0 ppm).

#### 3.4.2. Ophiobolins

The crude extract of a new species in *Aspergillus* section *Usti* (IBT 18591) was fractionated on a RP C_18_ (25 g, 33 mL) flash column with at gradient of: 15%–100% ACN in 20 min and flow rate 25 mL/min. Subsequently the active fraction was loaded on a diol flash column and eluted with 2 CV heptane, 2 CV heptanes/DCM (1:1), 2 CV DCM, 2 CV EtOAc, 2 CV MeOH. Ophiobolin K and 6-epiophiobolin K were isolated from the active fraction by semi-preparative HPLC on a Luna II, C_18_, 5 µm, 10 × 250 mm column by 70%–100% ACN in 25 min and flow rate 5 mL/min.

*Ophiobolin K*: White solid; [α]_589.3nm_: +161°; UV (ACN) λ_max_: 241 nm; HRMS *m/z* 384.2658 (M^+^calculated for C_25_H_36_O_3_, *m/z* 384.2659; 0.3 ppm); ^13^C- and ^1^H-NMR: see Tables S8 and S9, respectively.

*6-Epiophiobolin K*: White solid; [α]_589.3nm_: +53°; UV (ACN) λ_max_: 239 nm; HRMS *m/z* 384.2658 (M^+^calculated for C_25_H_36_O_3_, *m/z* 384.2659; 0.3 ppm); ^13^C- and ^1^H-NMR: see Tables S8 and S9, respectively.

The crude extract of *A. insuetus* (IBT 28266) was loaded on a diol flash column and eluted with 2 × 2 CV heptane, 2 CV heptanes/DCM (1:1), 2 CV DCM, 2 CV EtOAc, 2 CV MeOH. The active fraction was then loaded on a RP C_18_ flash column with at gradient of: 80%–100% ACN in 45 min and flow rate 40 mL/min. Ophiobolin U, ophiobolin H and 6-epiophiobolin N were isolated from the active fraction by semi-preparative HPLC on a Gemini, C_6_-Ph, 5 µm, 10 × 250 mm column. Ophiobolin U was purified by 55%–70% ACN in 25 min and flow rate 5 mL/min. Ophiobolin H and 6-epiophiobolin N were purified by 50% ACN isocratic in 15 min, then 50–60 min ACN in 15 min, and then up to 100% ACN in 5 min. ACN and MQ were added 50 ppm TFA.

*Ophiobolin U*: White solid; [α]_589.3nm_: +3°; UV (ACN) λ_max_: 242 nm; HRMS *m/z* 386.2813 (M^+^calculated for C_25_H_38_O_3_, *m/z* 386.2816; 0.5 ppm); ^1^H-NMR (800 MHz, CDCl_3_): δ 0.91 (2H, d, 6.7, H23), 0.99 s (3H, s, H22), 1.03 (1H, m, H1a), 1.26 (3H, s, H20), 1.38 (2H, m, H12), 1.55 (1H, m, H10), 1.58 (1H, m, H1b), 1.58 (1H, m, H13a), 1.74 (3H, s, H24), 1.78 (1H, m, H13b), 1.82 (3H, s, H25), 1.87 (1H, dd, 4.1, 15.1, H4a), 2.09 (1H, m, H14), 2.24 (1H, m, H9a), 2.30 (1H, m, H2), 2.68 (1H, dd, 7.9, 15.1, H4b), 2.72 (1H, m, H15), 2.89 (1H, dd, 8.5, 12.5, H9b), 3.02 d (1H, d, 9.6, H6), 4.91 (1H, dd, 4.7, 7.9, H5), 5.21 (1H, t, 10.0, H16), 6.00 (1H, m, H18), 6.03 (1H, m, H17), 6.94 t (1H, d, 8.5, H8), 9.26 (1H, s, H21); ^13^C-NMR (200 MHz, CDCl_3_): δ 18.3 (C24), 18.6 (C22), 20.6 (C23), 25.6 (C9), 26.3 (C20), 26.7 (C13), 26.7 (C25), 35.2 (C1), 35.9 (C15), 42.0 (C12), 44.1 (C11), 47.4 (C14), 50.5 (C6), 51.0 (C2), 53.4 (C4), 54.0 (C10), 73.1 (C5), 81.9 (C3), 120.2 (C18), 122.3 (C17), 135.9 (C19), 137.7 (C16), 142.1 (C7), 164.1 (C8), 198.1 (C21). Full dataset is found in Table S10.

*Ophiobolin H*: White solid; [α]_589.3nm_: +60°; UV (ACN) λ_max_: 241 nm; HRMS *m/z* 386.2817 (M^+^ calculated for C_25_H_38_O_3_, *m/z* 386.2816; −0.5 ppm); ^13^C- and ^1^H-NMR: see Tables S8 and S9, respectively.

*6-Epiophiobolin N*: White solid; [α]_589.3nm_: +10°; UV (ACN) λ_max_: 232 nm; HRMS *m/z* 368.2711 (M^+^ calculated for C_25_H_36_O_2_, *m/z* 368.2710; −0.2 ppm); ^13^C- and ^1^H-NMR: see Tables S8 and S9, respectively.

The crude extract of *A. calidoustus* (IBT 25726) was loaded on a diol flash column and eluted with 2 CV heptane, 2 CV heptanes/DCM (1:1), 2 CV DCM, 2 CV EtOAc, 2 CV MeOH. Ophiobolin C and 6-epiophiobolin G were isolated from the active fraction by semi-preparative HPLC on a Luna II, C_18_, 5 µm, 10 × 250 mm column. Ophiobolin C was purified by 80% ACN in MQ and 6-epiophiobolin G was purified by 80% MeOH in MQ both isocratic with a flow rate of 5 mL/min.

*Ophiobolin C*: White solid; [α]_589.3nm_: +298°; UV (ACN) λ_max_: 240 nm; HRMS *m/z* 386.2813 (M^+^ calculated for C_25_H_38_O_3_, *m/z* 386.2816; 0.7 ppm); ^13^C- and ^1^H-NMR: see Tables S8 and S9, respectively.

*6-Epiophiobolin G*: White solid; [α]_589.3nm_: +127°; UV (ACN) λ_max_: 234 nm; HRMS *m/z* 366.2553 (M^+^ calculated for C_25_H_34_O_2_, *m/z* 366.2554; 0.1 ppm); ^13^C- and ^1^H-NMR: see Tables S8 and S9, respectively.

### 3.5. CLL Cells, Cell Viability and Apoptosis Assays

Whole blood samples were obtained from patients that matched the standard diagnostic criteria for CLL after informed consent in accordance with the Declaration of Helsinki. All studies performed were approved by the ethics committee of the University of Ulm. Peripheral blood mononuclear cells (PBMC) were isolated by Ficoll density gradient and consisted of at least 80% CD5^+^CD19^+^ leukemic cells as determined by flow cytometry. For the initial screen, cocultures of HS-5 stromal cells and CLL cells were established as previously described [53]. For retesting of bio-active substances, CLL cells were cultured in conditioned media of HS-5 cells, which was harvested after 3–4 days of culture and 80% confluency and depleted of HS-5 cells and debris by centrifugation. CLL cells were seeded in duplicates at a density of 3 × 10^5^ cells/well in opaque-walled 96-well plates. Fractions or pure compounds were added in different concentrations and incubated for 24 h. 0.1% DMSO was used as a negative control. Cell viability was assessed using CellTiter-Glo^®^ assay (Promega, Madison, WI, USA) according to manufacturer’s protocol. Luminescence signals were recorded using a Mithras LB940 plate reader (Berthold Technologies, Bad Wildbad, Germany). Background signals of medium were subtracted from each well as described by Knudsen *et al.* [15].

Apoptotic cell death was detected by flow cytometry using Annexin V-phycoerythrin (PE) and 7-aminoactinomycin (7-AAD) staining kit (BD Biosciences, Heidelberg, Germany) as described by Seiffert *et al.* [12]. To confirm apoptosis induction, staining for active caspase 3 was performed after fixation and permeabilization of cells using BD Cytofix/Cytoperm^TM^ solution as described by the manufacturer by using PE-conjugated anti-active caspase 3 antibodies (clone C92-605, BD Biosciences). All flow cytometry analyses were carried out using a FACSCanto II flow cytometer equipped with FACSDiva software (BD Biosciences).

## 4. Conclusions

In conclusion, our combined bio-guided and dereplication based discovery approach has proven to be effective for fast dereplication and discovery of bioactive fungal natural products that target CLL cells. Comparative testing of active extracts on CLL cells as well as healthy cells identified compounds with general and selective bioactivity. The ophiobolin family showed high activity for CLL cells. The known ophiobolins A, B, C and K induced apoptosis in CLL cells with LC_50_ values of 1, 2, 8, and 4 nM, respectively with a lower bioactivity for healthy fibroblasts. The high activities for CLL cells were found only in ophiobolins with a hydroxy group at C3, an aldehyde at C21, and A/B-*cis* ring structure. In the remaining six bioactive extracts, the compounds responsible for the activity were tentatively identified by dereplication, and the activities towards CLL cells were verified by testing the pure compounds. The six active compounds, penicillic acid, viridicatumtoxin, calbistrin A, brefeldin A, emestrin A, and neosolaniol monoacetate, were all known and generally cytotoxic. In order to identify substances that are of therapeutic value for cancer cells, selective active substances need to be retested on a large cohort of cancer and healthy cells. In general, cytotoxic compounds are only suitable as anti-cancer pharmaceuticals if they selectively target the cancer cells and not healthy cells, or at least have a higher impact on the tumor cells. Activity optimization is well exemplified with these studies demonstrating the great potential of looking into the chemistry of closely related species to obtain more analogue compounds of a promising scaffold.

## Supplementary Materials

Supplementary materials can be accessed at: http://www.mdpi.com/1420-3049/18/12/14629/s1.
