# Supplementary material for: Bio-Activity and Dereplication-Based Discovery of Ophiobolins and Other Fungal Secondary Metabolites Targeting Leukemia Cells
Source: Molecules. 2013 Nov 26;18(12):14629–50. doi: 10.3390/molecules181214629 (PMC6290568; doi:10.3390/molecules181214629)

Supplementary Materials for

Bio-Activity and Dereplication Based Discovery of Ophiobolins and Other Fungal Secondary Metabolites Targeting Leukemia Cells

Contents

1. Strains tested in the initial screening against chronic lymphocytic leukemia (CLL)......... S4
2. Strains found active against chronic lymphocytic leukemia (CLL)................................. S6
3. Penicillic acid isolated from *Penicillium pulvillorum* (IBT 22393).................................. S8
4. Viridicatumtoxin isolated from *P. brasilianum* (IBT 22244).. S9
5. Emestrin A isolated from *Aspergillus* sp. (*Emericella*-like state) (IBT 22838)............... S10
6. Neosolaniol monoacetate isolated from *Fusarium compactum* (IBT 9034) S12
7. Ophiobolins.. S13
   - 7.1 Potential ophiobolin producing strains from the *Aspergilli* section *Usti*......................... S13
   - 7.2 NMR data for ophiobolin U, ophiobolin K, 6-epiophiobolin K, ophiobolin C, ophiobolin H, 6-epiophiobolin H, and 6-epiophiobolin G S14
   - 7.3 Ophiobolin U isolated from *A. insuetus* (IBT 28266) S16
   - 7.4 Ophiobolin H isolated from *A. insuetus* (IBT 28266) S18
   - 7.5 6-epiophiobolin N isolated from *A. insuetus* (IBT 28266).. S20
   - 7.6 Ophiobolin K isolated from a new sp. in the *Aspergillus* section *Usti* (IBT 18591)........ S22
   - 7.7 6-epiophiobolin K isolated from a new sp. in the *Aspergillus* section *Usti* (IBT 18591).. S24
   - 7.8 Ophiobolin C isolated from *A. calidoustus* (IBT 25726) S26
   - 7.9 6-epiophiobolin G isolated from *A. calidoustus* (IBT 25726).. S28
   - 7.10 Activity of ophiobolin A, B, C and K + 6-epiophiobolin K towards healthy fibroblasts (Wi-38) cells... S30
   - 7.11 Apoptosis induction in CLL cells by different ophiobolins. S31

List of Figures

Figure S1. ^{1}H-NMR spectrum of penicillic acid in DMSO-d_6 at 500 MHz.................................... S8
Figure S2. ^{1}H-NMR spectrum of viridicatumtoxin in DMSO-d_6 at 400 MHz................................ S9
Figure S3. ^{1}H-NMR spectrum of emestrin A in DMSO-d_6 at 500 MHz.. S10
Figure S4. ^{1}H-NMR spectrum of neosolaniol monoacetate in DMSO-d_6 at 500 MHz.................. S12
Figure S5. ^{1}H-NMR spectrum of ophiobolin U in $CDCl_3$.at 800 MHz.. S16
Figure S6. ^{13}C-NMR spectrum of ophiobolin U in $CDCl_3$.at 200 MHz. S16
Figure S7. ^{1}H-NMR spectrum of ophiobolin H in $CDCl_3$.at 800 MHz.. S18
Figure S8. ^{13}C-NMR spectrum of ophiobolin H in $CDCl_3$.at 200 MHz. S18
Figure S9. ^{1}H-NMR spectrum of 6-epiophiobolin N in $CDCl_3$.at 800 MHz. S20
Figure S10. ^{13}C-NMR spectrum of 6-epiophiobolin N in $CDCl_3$.at 200 MHz. S20
Figure S11. ^{1}H-NMR spectrum of ophiobolin K in $CDCl_3$.at 800 MHz.. S22
Figure S12. ^{1}H-NMR spectrum of 6-epiophiobolin K in $CDCl_3$.at 800 MHz. S24
Figure S13. ^{13}C-NMR spectrum of 6-epiophiobolin K in $CDCl_3$.at 200 MHz............................... S24
Figure S14. ^{1}H-NMR spectrum of ophiobolin C in $CDCl_3$.at 800 MHz... S26
Figure S15. ^{1}H-NMR spectrum of 6-epiophiobolin G in $CDCl_3$.at 800 MHz. S28
Figure S16. ^{13}C-NMR spectrum of 6-epiophiobolin G in $CDCl_3$.at 200 MHz. S28
Figure S17. Activity of ophiobolin A, B, C and K + 6-epiophioboln K towards healthy fibroblasts (Wi-38)... S30
Figure S18. CLL cells cultured in DMEM were treated for 24 hours with two different concentrations of ophiobolin A, ophiobolin B, ophiobolin C and ophiobolin K. In order to analyze apoptosis induction, cells of each well were divided into two parts and analyzed by flow cytometry using two different staining strategies. (a) cell survival was analyzed by gating on cells that were negative for Annexin V-phycoerythrin (PE) and 7-aminoactinomycin (7-AAD). Relative survival compared to DMSO control (0.1%) is depicted as mean values +SD of four independent CLL samples. (b) Caspase-3 activity is depicted as mean values +SD of four independent CLL samples relative to DMSO control (0.1%). S31

List of Tables

Table S1. Strains tested in the initial screening........ S4
Table S2. Strains found active in the initial screening........ S6
Table S3. ^{1}H and ^{13}C-NMR for penicillic acid in DMSO at 500 MHz for ^{1}H and 125 MHz for ^{13}C. S8
Table S4. ^{1}H-NMR for viridicatumtoxin in DMSO at 500 MHz. S9
Table S5. ^{1}H and ^{13}C-NMR for emestrin A in DMSO at 500 MHz for ^{1}H and 125 MHz for ^{13}C.. S11
Table S6. ^{1}H-NMR for neosolaniol monoacetate in DMSO at 500 MHz. S12
Table S7. *Aspergilli* section *Usti*. S13
Table S8. ^{13}C-NMR data for ophiobolin U, ophiobolin K, 6-epiophiobolin K, ophiobolin C, ophiobolin H, 6-epiophiobolin H, and 6-epiophiobolin G. (*125 MHz in dimethyl sulfoxide (DMSO)-d_6, †125 MHz in $CDCl_3$, or ‡200 MHz in $CDCl_3$, δ_C). S14
Table S9. ^{1}H-NMR data for ophiobolin U, ophiobolin K, 6-epiophiobolin K, ophiobolin C, ophiobolin H, 6-epiophiobolin H, and 6-epiophiobolin G. (*500 MHz in DMSO-d_6, †500 MHz in $CDCl_3$, or ‡ 800 MHz in $CDCl_3$, δ_H mult. (*J* (Hz))) S15
Table S10. ^{1}H and ^{13}C-NMR for ophiobolin U in $CDCl_3$.at 800 MHz for ^{1}H and 200 MHz for ^{13}C S17
Table S11. ^{1}H and ^{13}C-NMR for ophiobolin H in $CDCl_3$.at 800 MHz for ^{1}H and 200 MHz for ^{13}C S19
Table S12. ^{1}H and ^{13}C-NMR for 6-epiophiobolin N in $CDCl_3$.at 800 MHz for ^{1}H and 200 MHz for ^{13}C. S21
Table S13. ^{1}H and ^{13}C-NMR for ophiobolin K in DMSO-d_6.at 500 MHz for ^{1}H and 125 MHz for ^{13}C. S23
Table S14. ^{1}H and ^{13}C-NMR for 6-epiophiobolin K in DMSO-d_6.at 500 MHz for ^{1}H and 125 MHz for ^{13}C. S25
Table S15. ^{1}H and ^{13}C-NMR for ophiobolin C in $CDCl_3$.at 800 MHz for ^{1}H and 200 MHz for ^{13}C. 27
Table S16. ^{1}H and ^{13}C-NMR for 6-epiophiobolin G in $CDCl_3$.at 800 MHz for ^{1}H and 200 MHz for ^{13}C. S29

1. Strains tested in the initial screening against chronic lymphocytic leukemia (CLL)

Table S1. Strains tested in the initial screening.

Isolate number	Species	Isolate number	Species
21947	*Aspergillus cystallinus*	22516, 22523	*Penicillium faroense*
3234, 5265	*Aspergillus cavernicola*	21051	*Penicillium cluniae*
24813	*Aspergillus granulosus*	11843	*Penicillium decumbens*
24666	*Aspergillus conjunctus*	26291	*Penicillium bialowiezense*
10526, 18590	*Aspergillus caespitosus*	18329	*Penicillium brevicompactum*
22551	*Aspergillus diversus*	22244	*Penicillium brasillianum*
22564, 24812	*Aspergillus funiculosis*	22393	*Penicillium pulvillorum*
11054	*Aspergillus deflectus*	23856	*Penicillium svalbardense*
22568	*Aspergillus varians*	23667	*Penicillium lapatayae*
4537, 28161	*Aspergillus pseudoustus*	16536	*Penicillium alpinium*
14906, 23076	*Aspergillus subvesicolor*	14084	*Penicillium caseium*
10525	*Aspergillus neocaespitos*	16545	*Penicillium pinicola*
23282	*Aspergillus microcysticus*	22760	*Penicillium aquamarinum*
24752, 22153	*Aspergillus karnakataense*	22662	*Penicillium groenlandense*
26386, 22274	*Aspergillus janus*	24414	*Penicillium algidum*
17337	*Aspergillus aureolatus*	24411	*Penicillium jamesonlandense*
11835	*Aspergillus sydowii*	22544	*Penicillium eidiense*
10127	*Aspergillus quasivesicolor*	22356	*Penicillium monticola*
18591	New sp. in the *Aspergillus* section *Usti*	22663	*Penicillium floccosum*
21781, 22558	*Aspergillus brevis*	17760	*Penicillium artemision*
13670, 13691	*Aspergillus neopuniceus*	14073	*Penicillium wyomingense*
20587	*Aspergillus neoustus*	13954	*Penicillium minitum*
16756	*Aspergillus panamensis*	24420	*Penicillium rivulorum*
11860, 11847	*Aspergillus protuberus*	29798	*Penicillium diversicolor*
11821, 25061	*Aspergillus vesicolor*	16537	*Penicillium ribeum*
23160	*Aspergillus subsessilis*	16625	*Penicillium turcosum*
22554, 25041	*Aspergillus ustus*	22779	*Penicillium jugorum*
17674	*Aspergillus pseudoversicolor*	30014	*Penicillium galathea*
4332	*Aspergillus neovesicolor*	30013	*Penicillium nassarsuaquense*
18288	*Aspergillus arizonae*	fel 248, cml 832	*Pestalotiopsis*
13989	*Aspergillus aculeatus*	fel 64	*Arthrinium phaeospermum*
22838	*Aspergillus sp. (Emericella-like state)*	fel 192	*Ascochyta*

Table S1. *Cont.*

Isolate number	Species	Isolate number	Species
9194	*Cylindrocarpon olidum*	fell 01	*Acremonium*
8893	*Fusarium poae*	fel 240	*Paraconiothyrium*
8988	*Fusarium avenaceum*	cml 1707	*Nodulisporium*
8979, 9077, 9115	*Fusarium equiseti*	fel 89, fel 355, fel 364	*Phomopsis*
8977	*Fusarium tricinctum*	fel 302	*Cytospora*
8945, 7785	*Epicoccum nigrum*	fel 159	*Bipolaris sp.*
9034	*Fusarium compactum*	fel 06	*Glomerella cingulata*
9044	*Fusarium culmorum*	fel 307, fel 308	*Lecanicillium pasalliotae*
9063	*Fusarium cerealis*	fel 17, fel 42	*Paraconiothyrium sporulosum*
9085	*Fusarium oxysporum*	fel 05, fel 30, cml 1716	*Periconia*
9096	*Fusarium redolens*	fel 299A	*Curvularia*
9089	*Fusarium merismoides*	fel 142	*Tubercularia sp.*
9087	*Fusarium torulosum*	fel 315	*Verticillium leptobactum*
9086, 9206	*Fusarium solani*	cml 1692	*Lasiodiplodia theobromae*
9103	*Fusarium flocciferum*	cml 1709	*Collototrichum crassipes*
9112, 9121	*Fusarium pallidoroseum*	cml 1681	*Cuvularia prasadii*
9107	*Fusarium proliferatum*	cml 1671	*Libertella*
9117	*Fusarium verticillioides*	fel 58, cml 1702, cml 1703, cml 1690	*Microsphaeropsis*
1807	*Fusarium sambucinam*	cml 1670A	*Clonostachys*
9184	*Fusarium chlamydosporum*	cml 1693	*Spegazzinia deightonii*
9181	*Fusarium neohelle*	cml 1695	*Virgatospora echinofibrosa*
9182	*Fusarium neochlam*	fel 09, fel 241	*Paraconiothyrium brasiliense*

2. Strains found active against chronic lymphocytic leukemia (CLL)

Table S2. Strains found active in the initial screening.

Isolate number	Species	Maximum activity observed on media
9103	*Fusarium occiferum*	YES
9034	*Fusarium compactum*	YES
22838	*Aspergillus sp. (Emericella-like state)*	YES
13989	*Aspergillus aculeatus*	CYA
23105	*Aspergillus pseudovesicolor*	YES
3235	*Aspergillus cavernicola*	YES
18950	*Aspergillus caespitosus*	YES
22564	*Aspergillus funiculosis*	YES
22551	*Aspergillus diversus*	YES
14906	*Aspergillus subvesicolor*	CYA
23282	*Aspergillus microcysticus*	CYA
24752	*Aspergillus karnakataense*	YES
18951	New sp. in the *Aspergillus* section *Usti*	YES
20587	*Aspergillus neoustus*	YES
23160	*Aspergillus subsessilis*	CYA
26291	*Penicillium bialowienze*	YES
18329	*Penicillium brevicompactum*	YES
21051	*Penicillium cluniae*	YES
22393	*Penicillium pulvillorum*	YES
22244	*Penicillium brasilianum*	YES
11843	*Penicillium decumbens*	YES
18288	*Aspergillus arizonae*	CYA
16545	*Penicillium pinicola*	YES
22760	*Penicillium aquamarinum*	CYA
22523	*Penicillium faroense*	YES
22662	*Penicillium groenlandense*	ALK
24411	*Penicillium jamesonlandense*	CYA
22544	*Penicillium eidense*	YES
22356	*Penicillium monticola*	CYA
22663	*Penicillium occosum*	CYA
17760	*Penicillium artemision*	YES
29798	*Penicillium diversicolor*	YES
16537	*Penicillium ribeum*	YES
16625	*Penicillium turcosum*	ALK

Table S2. *Cont*.

Isolate number	Species	Maximum activity observed on media
22779	*Penicillium jugorum*	CYA
30014	*Penicillium galathea*	CYA
fel 248	*Pestalotiopsis*	MEA
fel 64	*Arthrinium phaeospermum*	MEA
fel 355	*Phomopsis*	MEA
fel 302	*Cytospora*	MEA
fel 159	*Bipolaris sp*	YES
fel 308	*Lecanicillium psalliotae*	YES
fel 240	*Paraconiothyrium brasiliense*	YES
fel 17	*Paraconiothyrium sporulosum*	YES
fel 05, fel 30 and cml 1716	*Periconia*	YES
fel 299A	*Curvularia*	YES
fel 142	*Tubercularia*	YES
cml 1692	*Lasiodiplodia theobromae*	YES
cml 1709	*Collototrichum crassipes*	YES
cml 1692	*Libertella*	YES
cml 1703, cml 1690	*Microphaeropsis*	YES
cml 1707	*Nodulisporium*	YES
cml 1670A	*Clonostachys*	YES
cml 1693	*Spegazzinia deightonii*	YES
cml 1695	*Virgatospora echinofibrosa*	YES

3. Penicillic acid isolated from *Penicillium pulvillorum* (IBT 22393)

Figure S1. ^{1}H-NMR spectrum of penicillic acid in DMSO-d_6 at 500 MHz

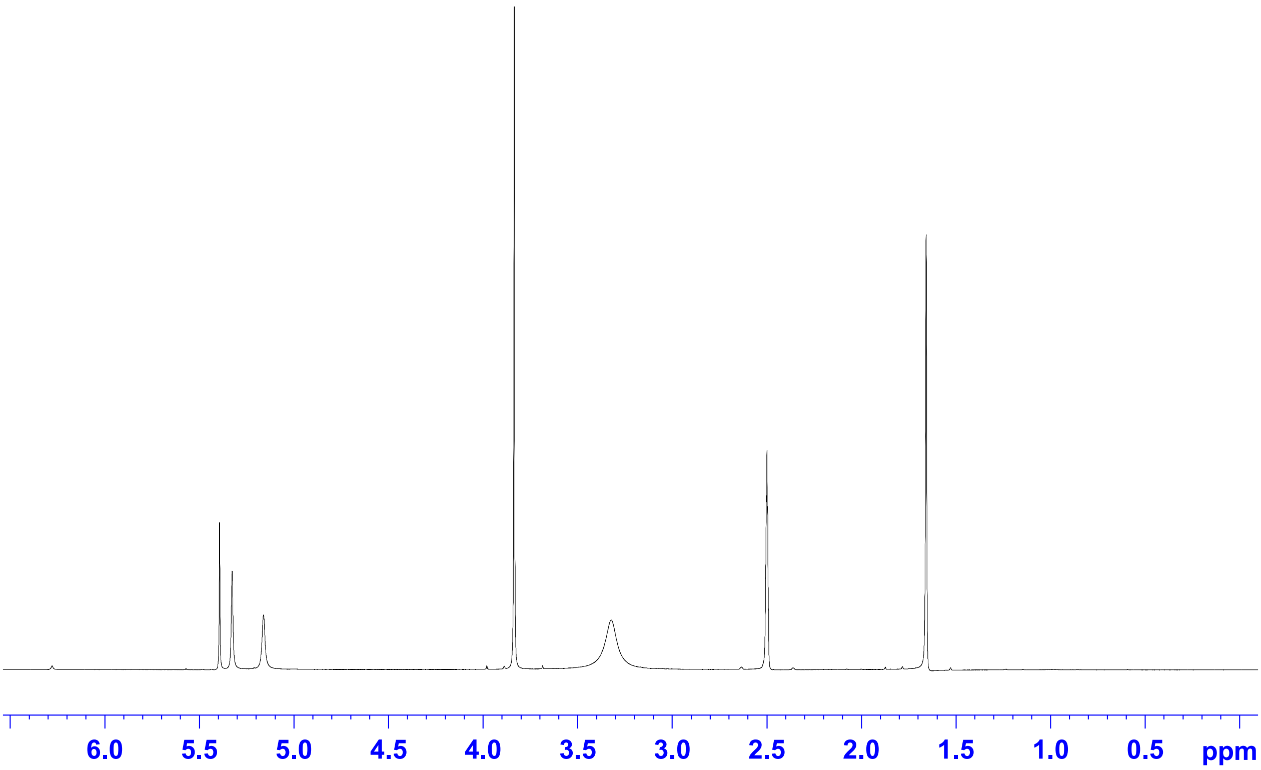

Table S3. ^{1}H and ^{13}C-NMR for penicillic acid in DMSO at 500 MHz for ^{1}H and 125 MHz for ^{13}C.

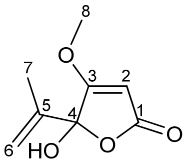

	δ_H mult. (J (Hz))	δ_C *
1		169.8
2	5.39 1 H, s	89.6
3		178.6
4		106.3
4-OH	3.33 1 H, br.s.	
5		140.4
6a	5.16 1 H, s	115.4
6b	5.33 1 H, br.s.	115.4
7	1.65 3 H, s	16.9
8	3.84 3 H, s	58.5

* ^{13}C-NMR chemical shifts determined from HSQC and HMBC experiments.

4. Viridicatumtoxin isolated from *P. brasilianum* (IBT 22244)

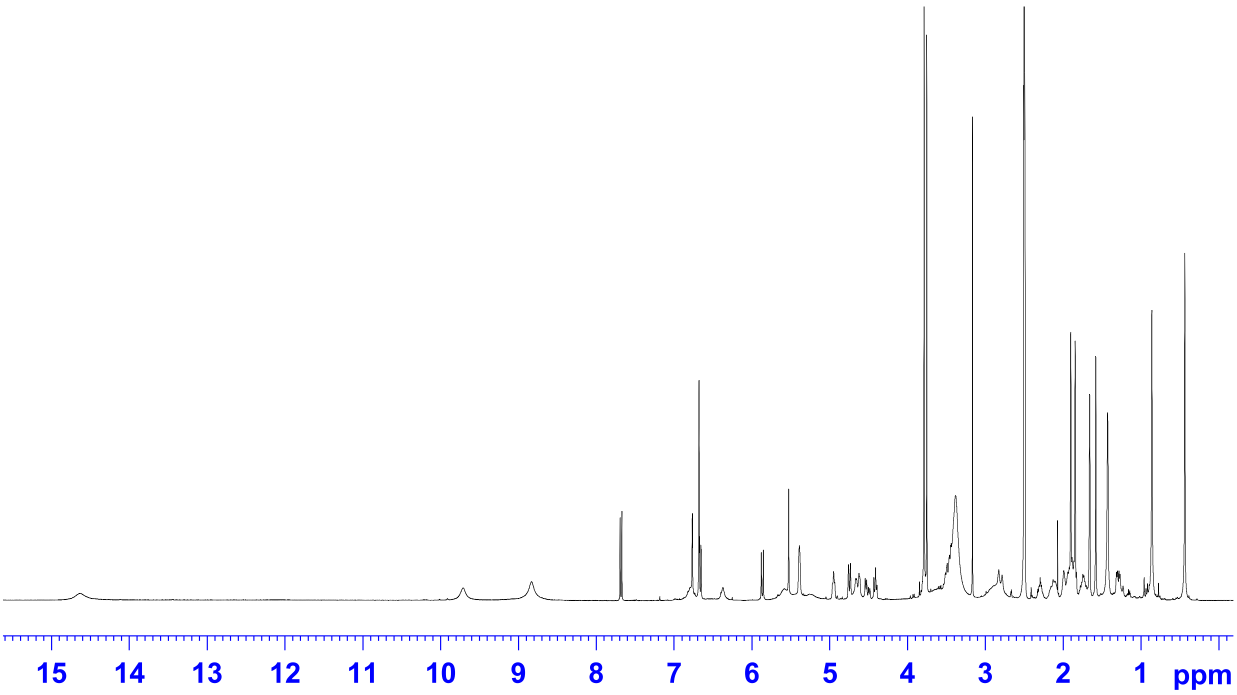

Figure S2. ^{1}H-NMR spectrum of viridicatumtoxin in DMSO-d_6 at 400 MHz.

Table S4. ^{1}H-NMR for viridicatumtoxin in DMSO at 500 MHz.

	δ_H mult. (J (Hz))
4α	2.55 (1H, m)
4β	2.81 (1H, m)
5	4.66 (1H, br.s.)
9	6.68 (1H, s)
14α	2.91 (1H, m)
14β	3.17 (1H, m)
17	5.40 (1H, br.s.)
18α	1.98 (1H, m)
18β	2.13 (1H, m)
19α	1.30 (1H, dd, 5.6, 12.8)
19β	1.75 (1H, m)
21	0.45 (3H, s)
22	0.87 (3H, s)
23	1.43 (3H, s)
24	3.79 (3H,s)
OH	5.59 (1H, br.s.)
OH	6.79 (1H, br.s.)
OH	8.84 (1H, br.s.)
OH	9.73 (1H, br.s.)
OH	14.64 (1H, br.s.)

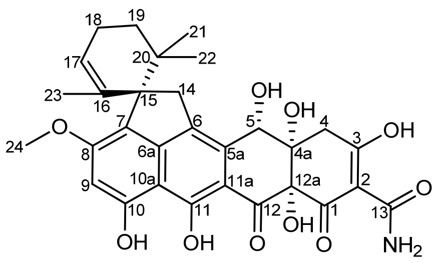

5. Emestrin A isolated from *Aspergillus* sp. (*Emericella*-like state) (IBT 22838)

Figure S3. ^{1}H-NMR spectrum of emestrin A in DMSO-d_6 at 500 MHz.

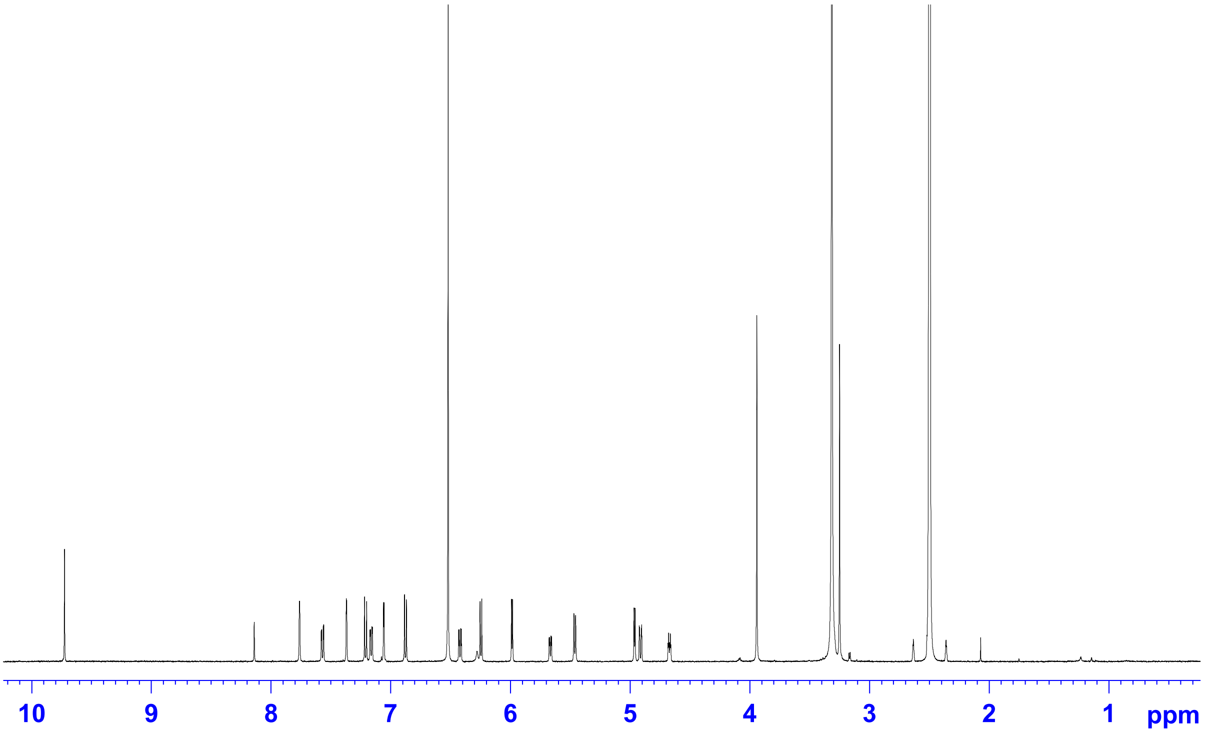

Table S5. ^{1}H and ^{13}C-NMR for emestrin A in DMSO at 500 MHz for ^{1}H and 125 MHz for ^{13}C.

	δ_H mult. (*J* (Hz))	δ_C *
1		80.8 ‡
2		164.2 †
3		80.8 ‡
4		164.2 †
5a	5.67 1 H, dd (2.5, 7.4)	59.8
5b		160.2
6	4.67 1 H, ddd (2.0, 2.5, 7.4)	74.6
7	4.91 1 H, dd (2.0, 8.5)	106.2
8	6.42 1 H, dd (2.5, 8.5)	137.1
10	7.06 1 H, d (2.5)	141.1
11	5.46 1 H, d (7.2)	72.6
11-OH	6.25 1 H, d (7.2)	
12	3.25 3 H, s	27.1
1'		127.2
2'	7.76 1 H, d (1.9)	122.5
3'		143.6
4'		149.0
4'-OH	9.73 1 H, s	
5'	6.88 1 H, d (8.3)	115.2
6'	7.16 1 H, dd (2.0, 8.3)	124.8
7'	4.96 1 H, d (4.6)	74.6
7'-OH	5.99 1 H, d (4.6)	
1"		145.6
2"		153.4
3"	7.21 1 H, d (8.6)	112.1
4"	7.57 1 H, dd (1.9, 8.6)	123.8
5"		122.0
6"	7.37 1 H, d (1.9)	120.0
7"		164.3
8"	3.94 3 H, s	55.7

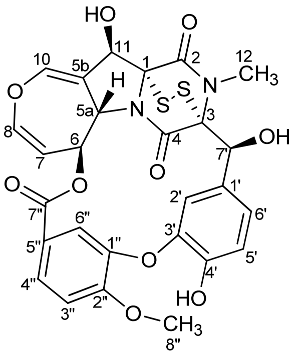

* ^{13}C-NMR chemical shifts determined from HSQC and HMBC experiments; ‡ Not possible to distinguish between C1 and C3; † Not possible to distinguish between C2 and C4.

6. Neosolaniol monoacetate isolated from *Fusarium compactum* (IBT 9034)

Figure S4. ^{1}H-NMR spectrum of neosolaniol monoacetate in DMSO-d_6 at 500 MHz.

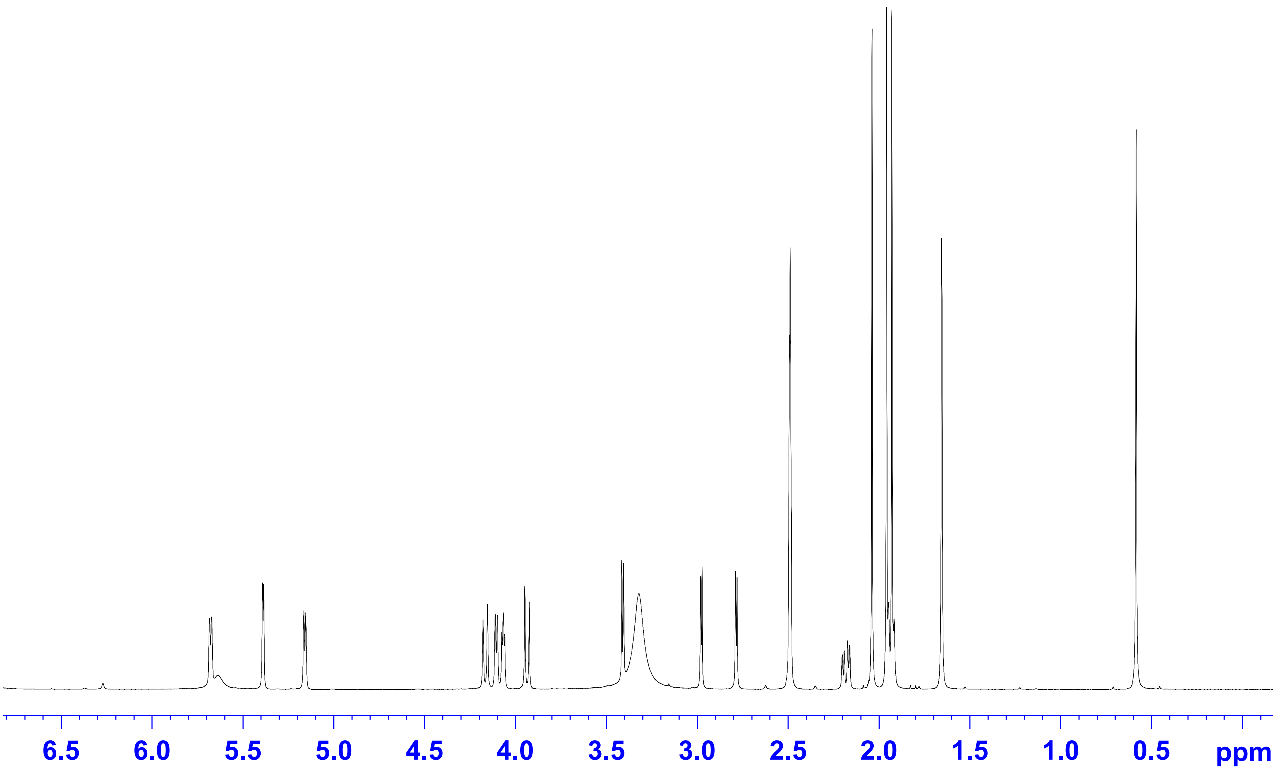

Table S6. ^{1}H-NMR for neosolaniol monoacetate in DMSO at 500 MHz.

	δ_H mult. (*J* (Hz))
2	3.41 1H, d (5.0)
3	4.07 1H, dd, (3.0; 5.0)
4	5.39 1H, d (3.2)
5	
6	
7a	2.18 1H, dd (5.3; 15.7)
7b	1.94 1H, m
8	5.15 1H, d, (5.3)
9	
10	5.67 1H, d (5.7)
11	4.1 1H, d (5.7)
12	
13a	2.78 1H, d (3.9)
13b	2.98 1H, d (3.9)
14	0.59 3H, s
15a	3.94 1H, d (12.2)
15b	4.16 1H, d (12.2)
16	1.66 3H, s
C=O C4	
C=O C8	
C=O C15	
CH3 C4	1.96 3H, s
CH3 C8	2.04 3H, s
CH3 C15	1.93 3H, s

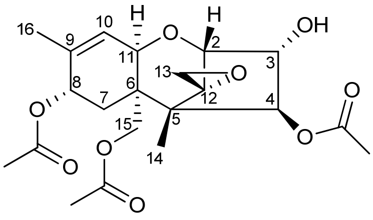

7. Ophiobolins

7.1 Potential ophiobolin producing strains from the *Aspergilli* section *Usti*

Table S7. *Aspergilli* section *Usti*.

IBT number	Species
4537	*Aspergillus ustus*
10619	
20587	
22554	
25041	
10524	*Aspergillus keveii*
24673	
24708	
28266	*Aspergillus insuetus*
28267	
13091	*Aspergillus calidoustus*
25726	

7.2 NMR data for ophiobolin U, ophiobolin K, 6-epiophiobolin K, ophiobolin C, ophiobolin H, 6-epiophiobolin H, and 6-epiophiobolin G

Table S8. ^{13}C-NMR data for ophiobolin U, ophiobolin K, 6-epiophiobolin K, ophiobolin C, ophiobolin H, 6-epiophiobolin H, and 6-epiophiobolin G. (* 125 MHz in dimethyl sulfoxide (DMSO)-d_6, † 125 MHz in $CDCl_3$, or ‡ 200 MHz in $CDCl_3$, δ_C).

Ophiobolin	U ‡	K *	6-epi-K *	C †	H ‡	6-epi-N ‡	6-epi-G †
**1**	35.2	34.5	41.0	36.0	35.7	45.6	45.8
**2**	51.0	49.2	49.6	50.8	50.9	49.3	49.2
**3**	81.9	76.2	74.7	76.6	80.2	179.8	178.1
**4**	53.4	53.9	54.7	54.6	50.8	130.0	130.2
**5**	73.1	217.0	216.1	217.5	116.0	209.6	208.2
**6**	50.5	48.3	48.2	48.4	52.8	49.9	50.0
**7**	142.1	140.6	141.5	141.5	138.5	140.1	139.9
**8**	164.1	157.6	159.9	163.8	123.6	157.3	158.1
**9**	25.6	24.3	30.0	24.7	25.0	31.0	30.9
**10**	54.0	53.0	43.1	53.4	55.0	43.1	43.8
**11**	44.1	43.3	44.9	43.6	43.6	45.0	45.4
**12**	42.0	42.2	44.4	42.6	43.0	44.5	44.3
**13**	26.7	25.4	27.1	22.8	26.8	27.1	27.8
**14**	47.4	46.2	51.4	45.2	47.2	51.1	52.1
**15**	35.9	34.4	31.9	32.7	35.5	31.8	32.6
**16**	137.7	136.9	136.0	36.8	138.0	37.1	135.7
**17**	122.3	121.7	123.3	26.0	121.7	25.6	124.0
**18**	120.2	119.9	120.1	124.3	120.4	124.4	120.0
**19**	135.9	134.6	135.3	131.0	135.2	131.6	136.6
**20**	26.3	25.7	25.2	25.4	25.4	17.4	17.3
**21**	198.1	193.1	194.7	195.9	71.5	193.0	193.0
**22**	18.6	18.2	22.8	19.0	18.7	23.1	22.9
**23**	20.6	19.6	21.1	16.4	20.4	18.6	21.3
**24**	18.3	18.0	17.9	17.6	18.2	17.7	18.2
**25**	26.7	25.9	26.1	25.6	26.6	25.7	26.5

Table S9. ^{1}H-NMR data for ophiobolin U, ophiobolin K, 6-epiophiobolin K, ophiobolin C, ophiobolin H, 6-epiophiobolin H, and 6-epiophiobolin G. (* 500 MHz in DMSO-d_6, † 500 MHz in $CDCl_3$, or ‡ 800 MHz in $CDCl_3$, δ_H mult. (J (Hz))).

Ophiobolin	U ‡	K *	6-epi-K *	C †	H ‡	6-epi-N ‡	6-epi-G †
**1a**	1.03 m	1.14 m	1.58 m	1.26 m	1.36 m	1.18 m	1.15 m
**1b**	1.58 m	1.58 m	1.65 m	1.81 m	1.42 m	2.04 dd (3.7; 13.2)	2.03 m
**2**	2.30 m	2.25 m	1.91 m	2.38 m	2.26 m	2.71 m	2.66 m
**3-OH**			6.51 br.s.				
**4a**	1.87 dd (4.1, 15.1)	2.35 m	2.21 m	2.49 m	2.10 m	6.11 s	6.04 s
**4b**	2.68 dd (7.9, 15.1)	2.50 m	2.77 d (16.0)	2.80 m	2.18 m		
**5**	4.91 dd (4.7, 7.9)						
**6**	3.02 d (9.6)	3.22 d (9.8)	3.04 d (10.8)	3.26 m	3.17 d (9.8)	3.54 d (3.7)	3.40 m
**8**	6.94 t (8.5)	7.02 t (8.5)	6.96 m	7.21 m	5.64 br.s.	6.86 dd (2.0; 6.3)	6.80 m
**9a**	2.24 m	2.04 m	2.27 m	2.31 m	1.70 m	2.25 m	2.20 m
**9b**	2.89 dd (8.5, 12.5)	2.69 m	2.65 m	2.45 m	2.50 dd (8.6; 13.8)	2.71 m	2.93 m
**10**	1.55 m	1.56 m	2.46 m	1.67 m	1.60 m	2.72 m	2.63 m
**12a**	1.38 m	1.33 m	1.38 m	1.41 m	1.40 dd (7.6; 11.7)	1.42 m	1.43 m
**12b**		1.35 m	1.44 m	1.44 m	1.57 m	1.51 m	1.52 m
**13a**	1.58 m	1.54 m	1.22 m	1.46 m	1.54 m	1.23 m	1.25 m
**13b**	1.78 m	1.69 m	1.57 m	1.55 m	1.76 m	1.58 m	1.67 m
**14**	2.09 m	2.09 m	1.87 m	2.36 m	2.05 m	1.75 m	1.89 m
**15**	2.72 m	2.72 m	2.61 m	1.65 m	2.68 m	1.42 m	2.55 m
**16a**	5.21 t (10.0)	5.23 t (9.5)	5.25 t (9.3)	1.18 m	5.20 m	0.99 m	5.11 t (1.3)
**16b**				1.24 m		1.45 m	
**17a**	6.03 m	5.99 m	6.11 m	1.95 m	5.99 m	1.94 m	6.10 m
**17b**				2.00 m		2.07 m	
**18**	6.00 m	6.04 m	6.09 m	5.09 m	5.98 m	5.12 t (7.0)	6.00 m
**20**	1.26 s	1.19 s	1.26 s	1.36 s	1.24 s	2.10 s	2.06 s
**21a**	9.26 s	9.14 s	9.12 s	9.23 s	4.48 br.s.	9.30 s	9.26 s
**21b**					4.59 d (12.2)		
**22**	0.99 s	0.91 s	0.77 s	0.90 s	0.90 s	0.86 m	0.85 s
**23**	0.91 d (6.7)	0.85 d (6.6)	0.92 d (6.6)	0.78 d (6.8)	0.88 d (6.7)	0.91 d (6.4)	0.97 d (6.8)
**24**	1.74 s	1.68 s	1.70 s	1.61 s	1.73 s	1.61 s	1.76 s
**25**	1.82 s	1.76 s	1.79 s	1.69 s	1.80 s	1.69 s	1.83 s

7.3 Ophiobolin U isolated from *A. insuetus* (IBT 28266)

Figure S5. ^{1}H-NMR spectrum of ophiobolin U in $CDCl_3$ at 800 MHz.

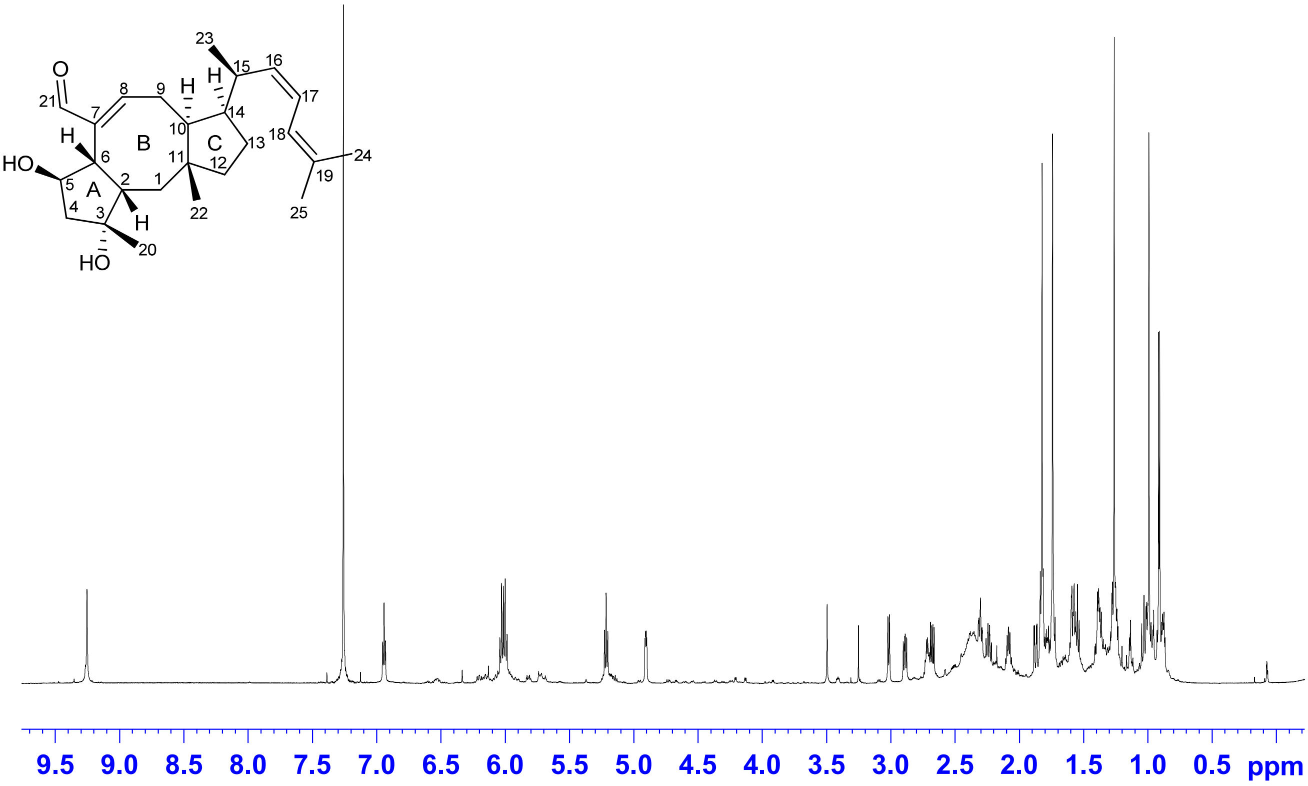

Figure S6. ^{13}C-NMR spectrum of ophiobolin U in $CDCl_3$ at 200 MHz.

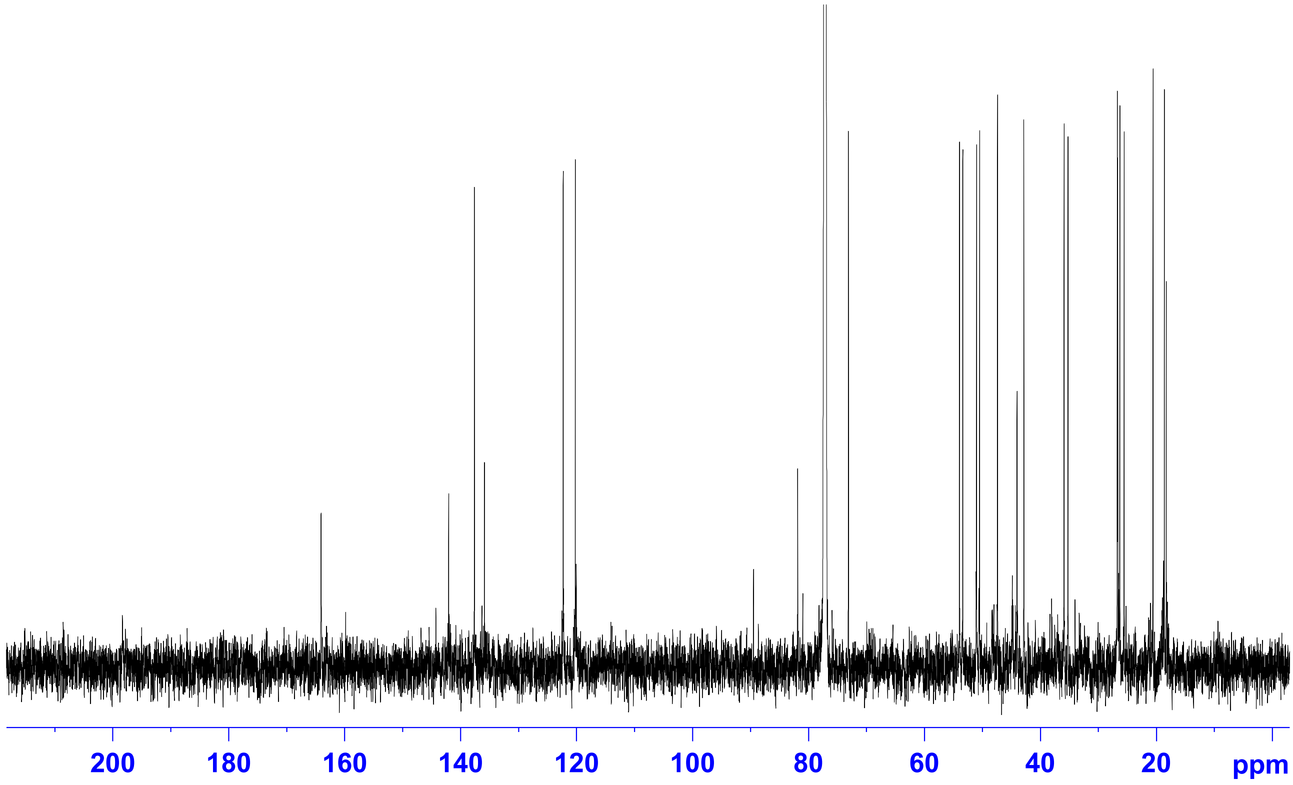

Table S10. ^{1}H- and ^{13}C-NMR for ophiobolin U in $CDCl_3$ at 800 MHz for ^{1}H and 200 MHz for [13]C.

	δ_H mult. (*J* (Hz))	δ_C	HMBC connectivities	NOE connectivities
1a	1.03 (1H, m)	35.2	2, 3, 10, 11, 22	1b
1b	1.58 (1H, m)	35.2	3, 10, 12	1a, 2, 15, 20, 22
2	2.30 (1H, m)	51.0	1, 3, 6, 7	6, 20, 22
3		81.9		
4a	1.87 (1H, dd, 4.1, 15.1)	53.4	3, 5	2, 4b, 20
4b	2.68 (1H, dd, 7.9, 15.1)	53.4	2, 3, 6	4a, 5
5	4.91 (1H, dd, 4.7, 7.9)	73.1	3, 6, 7	4b, 6
6	3.02 d (1H, d, 9.6)	50.5	2, 3, 5, 7, 8, 21	2, 5, 9a, 22
7		142.1	-	-
8	6.94 t (1H, d, 8.5)	164.1	6, 9, 21	9a, 9b, 10, 21
9a	2.24 (1H, m)	25.6	7, 8, 10, 11	6, 8, 9b, 22
9b	2.89 (1H, dd, 8.5, 12.5)	25.6	7, 8, 10, 11, 14	8, 9a, 10, 14, 15, 16
10	1.55 (1H, m)	54.0	8, 9, 11, 14, 15, 22	8, 9b, 14
11		44.1	-	-
12	1.38 (2H, m)	42.0	10, 11, 13, 14, 22	13a, 13b, 22
13a	1.58 (1H, m)	26.7	11, 14, 15	12, 13b, 22, 23
13b	1.78 (1H, m)	26.7	10, 11, 14, 15	12, 13a, 14
14	2.09 (1H, m)	47.4	10, 11, 13, 15, 16, 23	9b, 10, 13b, 15, 16, 23
15	2.72 (1H, m)	35.9	14, 16, 17, 23	9b, 1b, 14, 16, 18, 22, 23
16	5.21 (1H, t, 10.0)	137.7	14, 15, 18, 23	9b, 14, 15, 17, 23
17	6.03 (1H, m)	122.3	15, 19	16, 24
18	6.00 (1H, m)	120.2	24, 25	15, 25
19		135.9		
20	1.26 (3H, s)	26.3	2; 3; 4	1b, 2, 4a
21	9.26 (1H, s)	198.1	6, 7, 8	5, 8
22	0.99 s (3H, s)	18.6	1, 10, 11, 14	1b, 2, 6, 9a, 12, 13a, 15
23	0.91 (2H, d, 6.7)	20.6	14, 15, 16	13a, 14, 15, 16
24	1.74 (3H, s)	18.3	18, 19, 25	17
25	1.82 (3H, s)	26.7	18, 19, 24	18

7.4 Ophiobolin H isolated from *A. insuetus* (IBT 28266)

Figure S7. ^{1}H-NMR spectrum of ophiobolin H in $CDCl_3$ at 800 MHz.

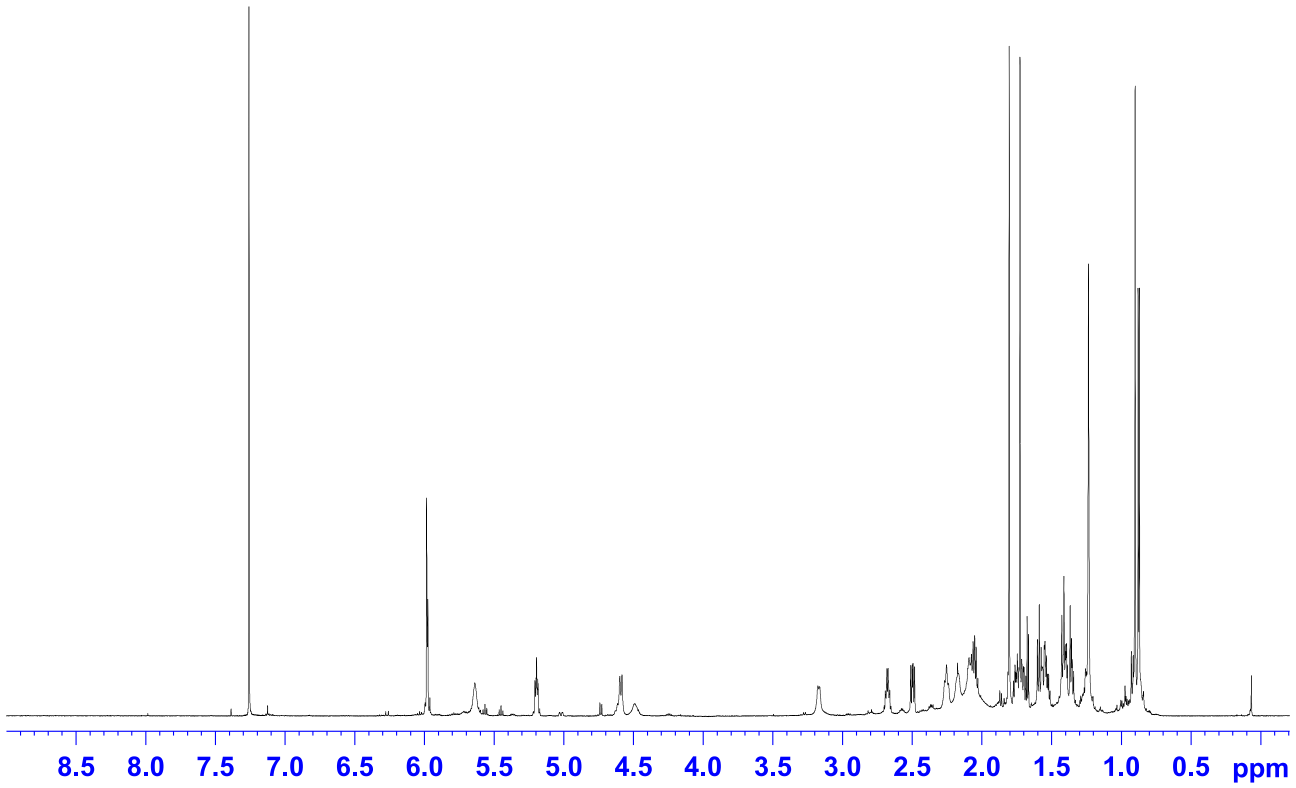

Figure S8. ^{13}C-NMR spectrum of ophiobolin H in $CDCl_3$ at 200 MHz.

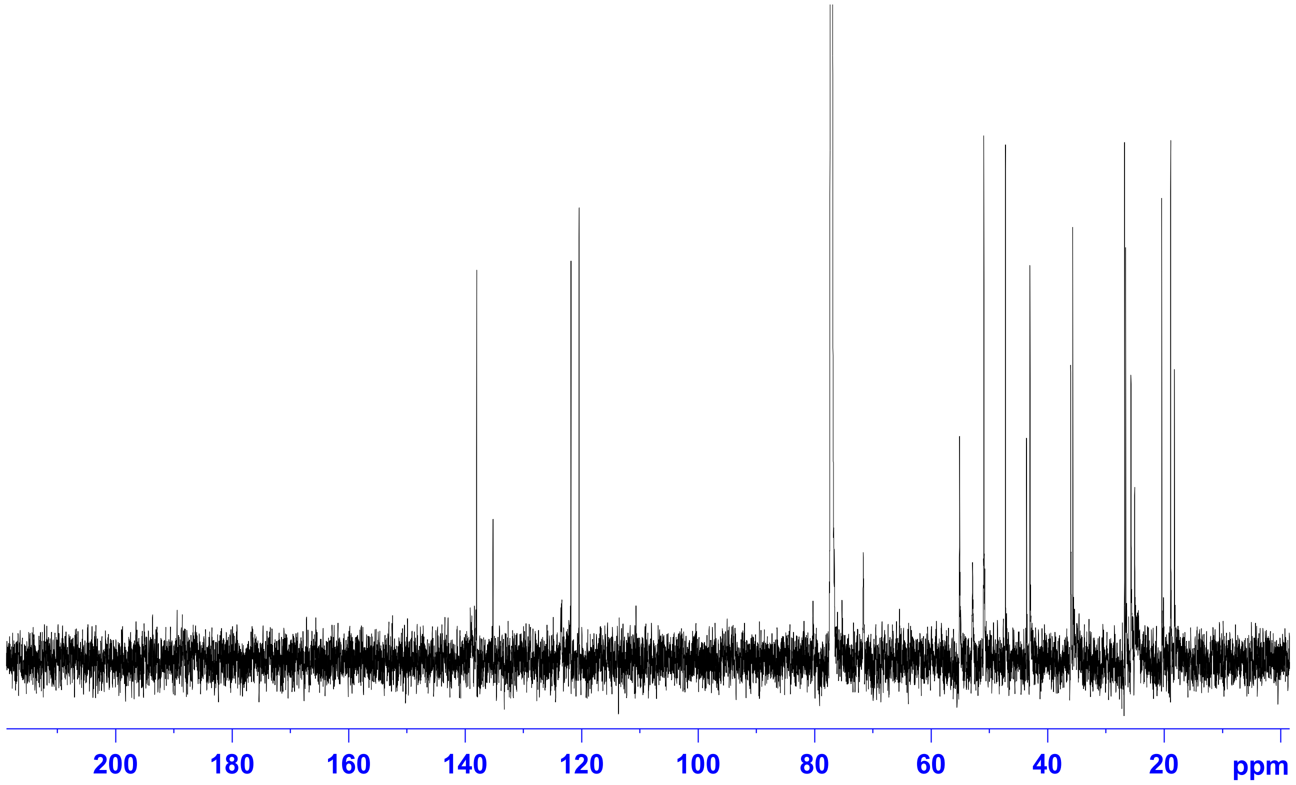

Table S11. ^{1}H- and ^{13}C-NMR for ophiobolin H in $CDCl_3$ at 800 MHz for ^{1}H and 200 MHz for ^{13}C.

	δ_H mult. (*J* (Hz))	δ_C	HMBC connectivities	NOE connectivities
1a	1.36 m	35.7	2, 6, 10, 11, 22	1b
1b	1.42 m	35.7		1a, 2, 8, 20
2	2.26 m	50.9		1b, 6, 20, 22
3		80.2		
4a	2.10 m	50.8		4b, 20
4b	2.18 m	50.8		4a
5		116.0		
6	3.17 d (9.8)	52.8		2, 9a, 22
7		138.5		
8	5.64 br.s.	123.6		1b, 9b
9a	1.70 m	25.0	8, 10, 11	6, 9b, 22
9b	2.50 dd (8.6; 13.8)	25.0	7, 8, 10, 11, 14	8, 9a, 10, 15, 16
10	1.60 m	55.0	8, 9, 11, 14, 15, 22	9b, 14
11		43.6		
12a	1.40 dd (7.6; 11.7)	43.0	10, 11, 14, 22	12b, 13a, 22
12b	1.57 m	43.0	13, 15	12a, 13b
13a	1.54 m	26.8	12, 14, 15	12a, 13b, 23
13b	1.76 m	26.8	10, 11, 14	12b, 13a, 14
14	2.05 m	47.2	10, 11, 13, 15, 16, 23	10, 13b, 16, 23
15	2.68 m	35.5	14, 16, 23	9b, 18, 22
16	5.20 m	138.0	18	9b, 14, 17, 23
17	5.99 m	121.7	15, 19	16, 24
18	5.98 m	120.4	16, 24, 25	15, 23, 25
19		135.2		
20	1.24 s	25.4	2, 3	1b, 2, 4a
21a	4.48 br.s.	71.5		
21b	4.59 d (12.2)	71.5		
22	0.90 s	18.7	1, 10, 11	2, 6, 9a, 12a, 15
23	0.88 d (6.7)	20.4	14, 15, 16	13a, 14, 16, 18
24	1.73 s	18.2	18, 19, 25	17
25	1.80 s	26.6	18, 19, 24	18

7.5 6-epiophiobolin N isolated from *A. insuetus* (IBT 28266)

Figure S9. ^{1}H-NMR spectrum of 6-epiophiobolin N in $CDCl_3$ at 800 MHz.

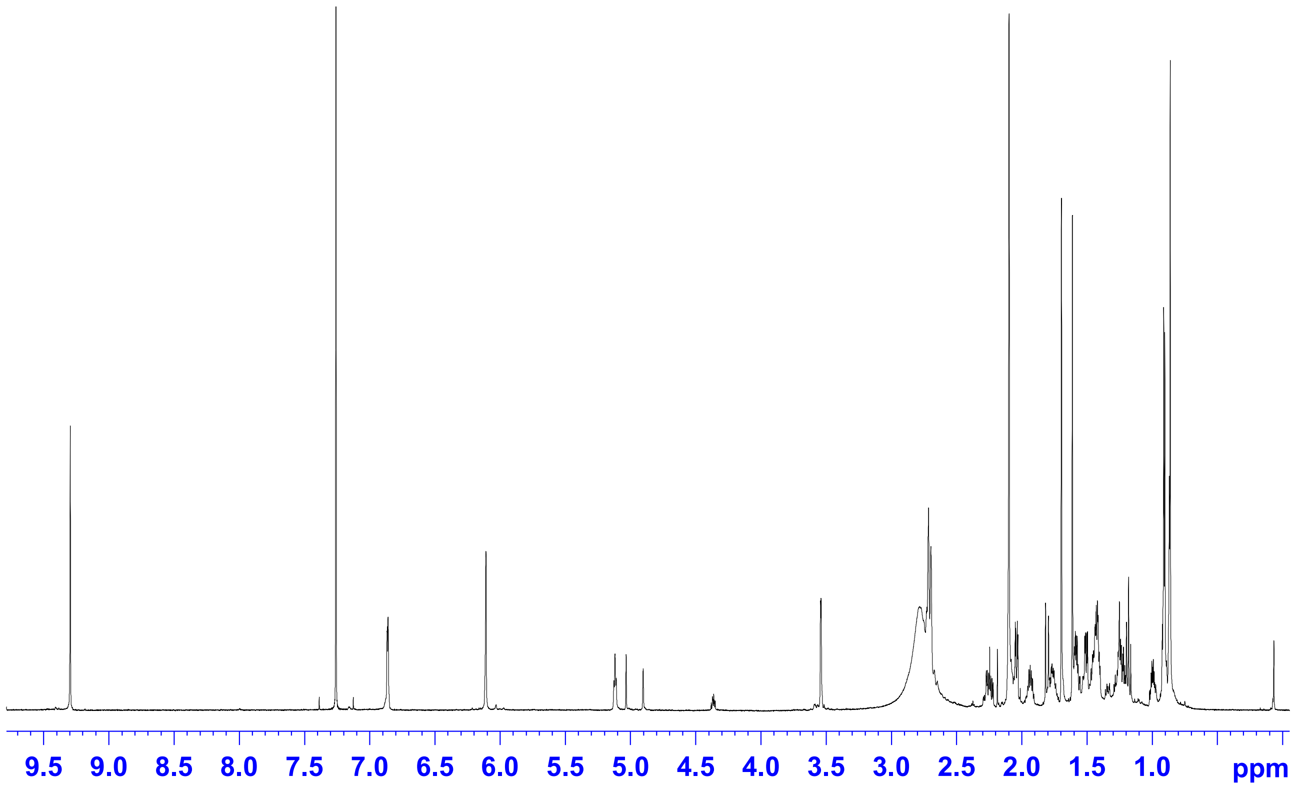

Figure S10. ^{13}C-NMR spectrum of 6-epiophiobolin N in $CDCl_3$ at 200 MHz.

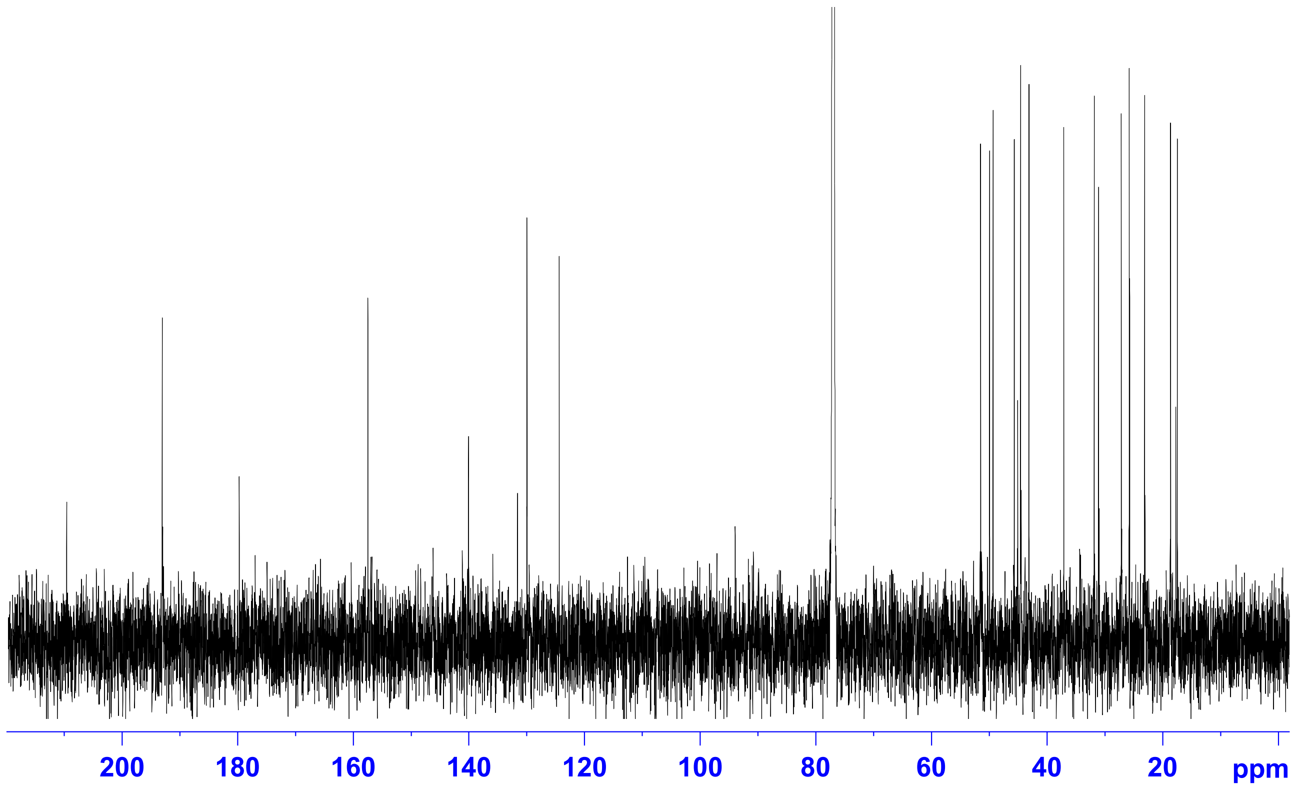

Table S12. ^{1}H and ^{13}C NMR for 6-epiophiobolin N in $CDCl_3$.at 800 MHz for ^{1}H and 200 MHz for ^{13}C.

	δ_H mult. (J (Hz))	δ_C	HMBC connectivities	NOE connectivities
1a	1.18 m	45.6	2; 3; 10; 12; 22	1b, 6, 10, 12a/15 *
1b	2.04 dd (3.7; 13.2)	45.6	2, 6, 10, 11	1a, 10, 12a/15 *, 12b, 22
2	2.71 m	49.3	8, 11	8. 9a, 16b, 20, 22
3		179.8		
4	6.11 s	130.0	3; 5; 6; 20	20
5		209.6		
6	3.54 d (3.7)	49.9	1; 2; 5; 7; 8; 21	1a; 2; (8); 9a, 10
7		140.1		
8	6.86 dd (2.0; 6.3)	157.3	6; 10; 21	2/9b **, (6), 9a, 21, 22
9a	2.25 m	31.0	7; 8; 10	2/9b **, 6, 8, 12a, 22
9b	2.71 m	31.0	8, 11	8. 9a, 12a/15 *, 20, 22
10	2.72 m	43.1	1, 7, 13, 14, 22	1a, 1b, 6, 14, 16b
11		45.0		
12a	1.42 m	44.5	1, 13, 22	1a, 1b, 9b, 12b, 13b, 14, 17a, 23
12b	1.51 m	44.5	10; 11; 14; 22	1b; 12a/15 *; 13a; 13b; 22
13a	1.23 m	27.1	12; 14; 15	12b; 13b; 22; 23
13b	1.58 m	27.1	10; 11	13a; 14, 12a/15 *; 23
14	1.75 m	51.1		9b, 10, 12a/15 *, 13b
15	1.42 m	31.8		1a, 1b, 9b, 12b, 13b, 14, 17a, 23
16a	0.99 m	37.1	15; 17; 18; 23	10; 17b; 18
16b	1.45 m	37.1	23	2/9b **; 10, 16a
17a	1.94 m	25.6	16; 18; 19	12a/15 *; 17b; 23
17b	2.07 m	25.6		16a; 17a; 23
18	5.12 t (7.0)	124.4	24; 25	15; 16a; 25
19		131.6		
20	2.10 s	17.4	2, 3, 4	2/9b **; 4
21	9.30 s	193.0	6, 7	8
22	0.86 m	23.1	1; 10; 12	1b; 2/9b **; 8; 9a; 12b; 13a
23	0.91 d (6.4)	18.6	14; 15; 16	10; 13a; 13b; 15; 16a; 17a; 17b
24	1.61 s	17.7	18; 19; 25	25
25	1.69 s	25.7	18; 19; 24	18; 24

* Not possible to distinguish between H12a and H15; ** Not possible to distinguish between H2 and H9b.

7.6 Ophiobolin K isolated from a new sp. in the *Aspergillus* section *Usti* (IBT 18591)

Figure S11. ^{1}H-NMR spectrum of ophiobolin K in $CDCl_3$ at 800 MHz.

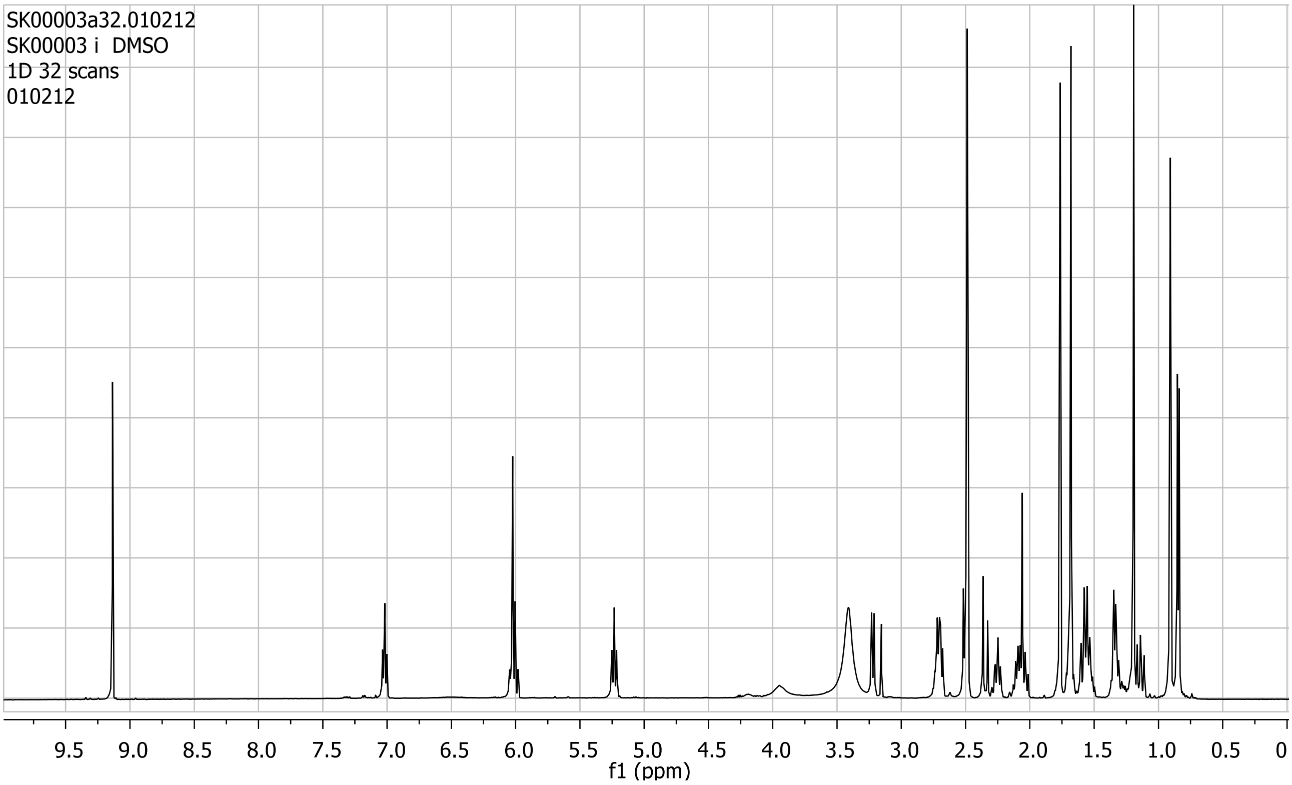

Table S13. ^{1}H and ^{13}C-NMR for ophiobolin K in DMSO-d_6 at 500 MHz for ^{1}H and 125 MHz for [13]C.

	δ_H mult. (*J* (Hz))	δ_C *	HMBC connectivities	NOE connectivities
1a	1.14 m	34.5	6, 10, 11, 22	
1b	1.58 m		6, 10, 11	2, 12a, 20
2	2.25 m	49.2	1, 4, 6, 7	1b, 4a, 6, 20, 22
3		76.2		
4a	2.35 m	53.9	3, 5, 20	2, 20
4b	2.50 m		2, 3, 5	
5		217.0		
6	3.22 d (9.8)	48.3	2, 3, 5, 7, 8, 21	2, 9a, 22
7		140.6		
8	7.02 t (8.5)	157.6	6, 9, 21	9b, 10, 21
9a	2.04 m	24.3	7, 8, 10, 11	6, 22
9b	2.69 m		7, 8, 10, 11, 14	8, 10, 16
10	1.56 m	53.0	1, 8, 9, 11, 14, 22	8, 9b, 12
11		43.3		
12a	1.33 m	42.2	10, 11, 13, 14, 22	1b, 13b, 22
12b	1.35 m			
13a	1.54 m	25.4	11, 15	14, 22, 23, 24
13b	1.69 m		11	12a, 14
14	2.09 m	46.2	10, 11, 13, 15, 16, 23	13a/b, 15, 16 , 23
15	2.72 m	34.4	16, 17, 23	14, 18, 22, 23
16	5.23 t (9.5)	136.9	14, 15, 18, 23	9b, 14, 17, 23
17	5.99 m	121.7	15, 18	16, 24
18	6.04 m	119.9	16, 19, 24, 25	15, 23, 25
19		134.6		
20	1.19 s	25.7	2, 3, 4	1b, 2, 4a
21	9.14 s	193.1	6, 7, 8	8
22	0.91 s	18.2	1, 10, 11	2, 6, 9a, 12a, 13a,15
23	0.85 d (6.6)	19.6	14, 15, 16	13a, 14, 15, 16, 18
24	1.68 s	18.0	18, 19, 25	13a, 17, 25
25	1.76 s	25.9	18, 19, 24	18, 24

* ^{13}C NMR chemical shifts determined from HSQC and HMBC experiments.

7.7 6-Epiophiobolin K isolated from a new sp. in the *Aspergillus* section *Usti* (IBT 18591)

Figure S12. ^{1}H-NMR spectrum of 6-epiophiobolin K in $CDCl_3$ at 800 MHz.

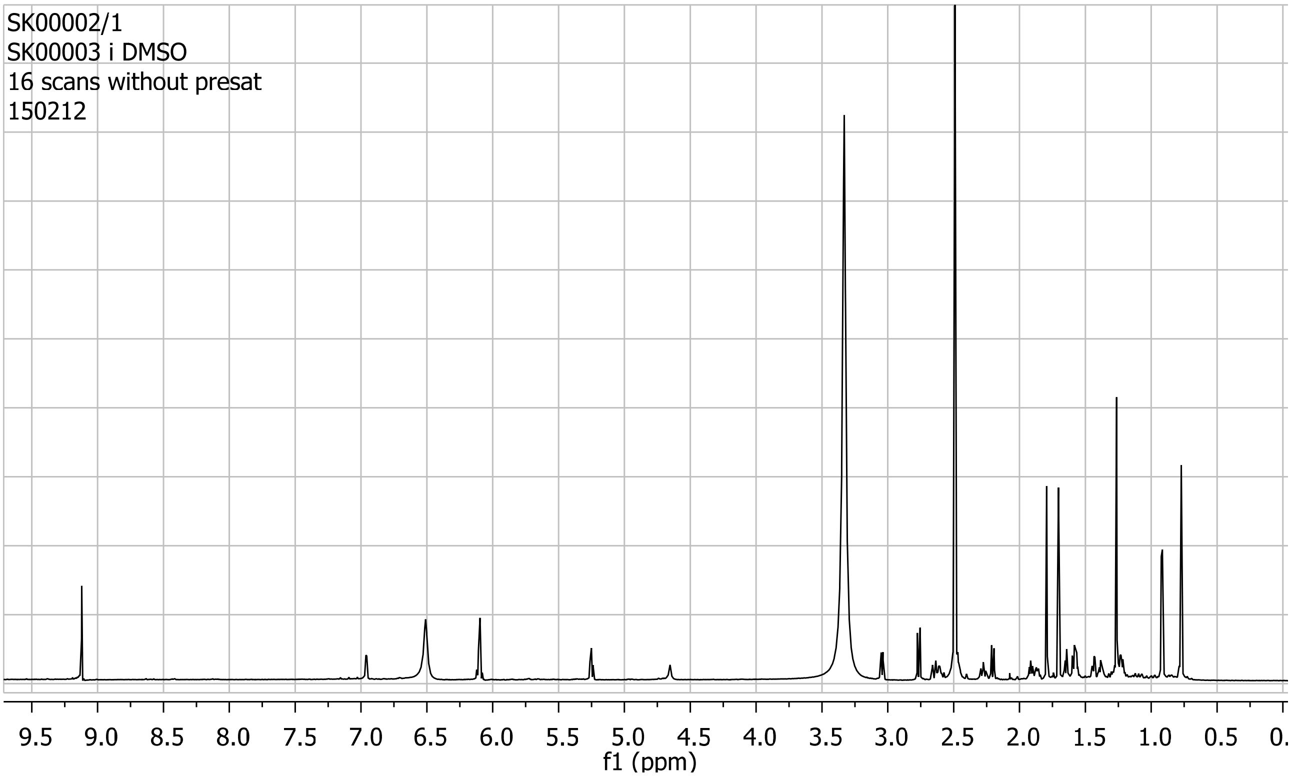

Figure S13. ^{13}C-NMR spectrum of 6-epiophiobolin K in $CDCl_3$ at 200 MHz.

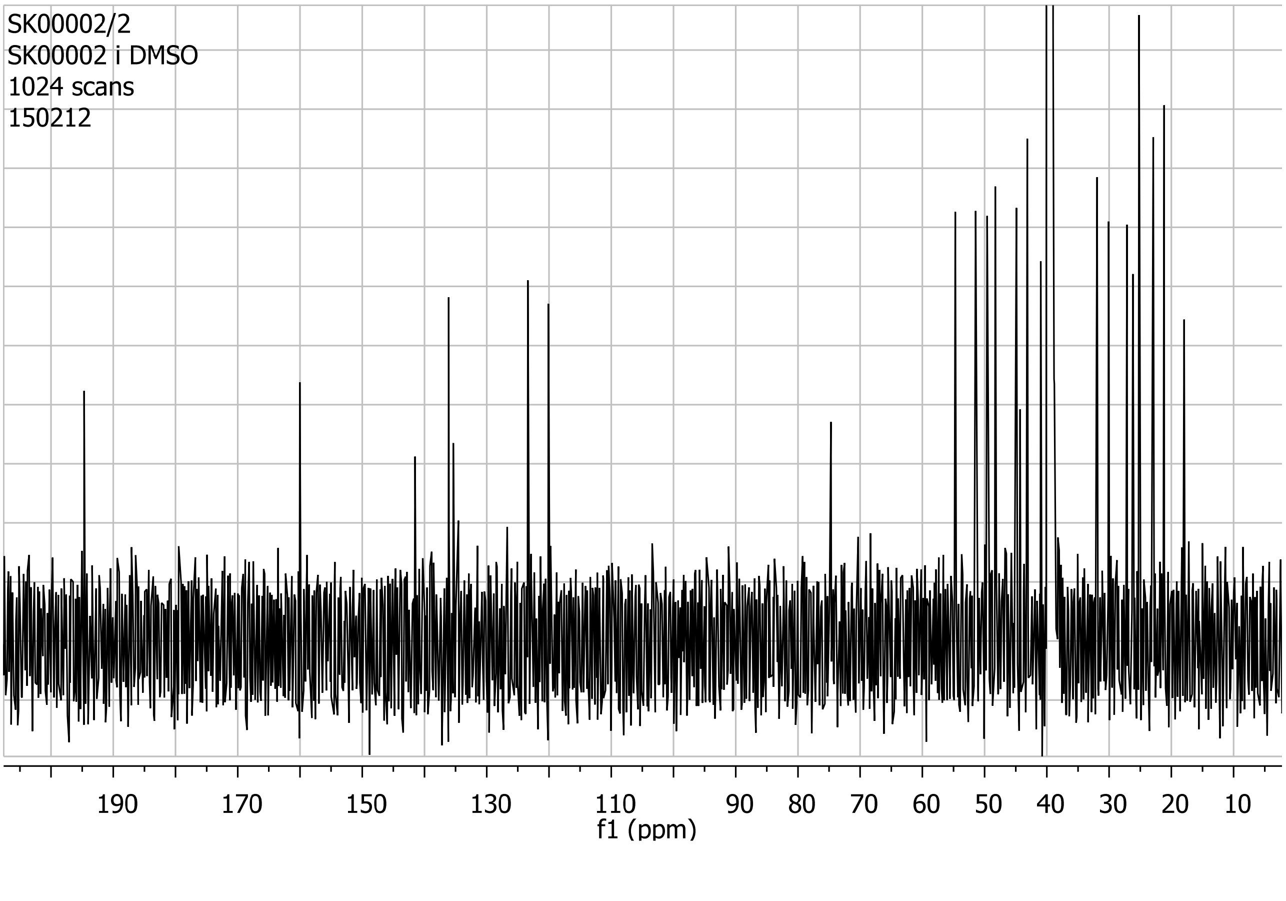

Table S14. ^1H and ^{13}C-NMR for 6-epiophiobolin K in DMSO-d_6 at 500 MHz for ^1H and 125 MHz for ^{13}C.

	δ_H mult. (J (Hz))	δ_C	HMBC connectivities	NOE connectivities
1a	1.58 m	41.0	2, 6, 10, 22	6, 12a
1b	1.65 m			2, 20, 22
2	1.91 m	49.6	6, 7	1a/b , 4b, 20, 22
3		74.7		
3-OH	6.51 br.s.			
4a	2.21 m	54.7	2, 3, 5	20 (w)
4b	2.77 d (16.0)		3, 5, 20	2, 20
5		216.1	-	
6	3.04 d (10.8)	48.2	1, 2, 5, 7, 21	1a, 2 (w), 10
7		141.5		
8	6.96 m	159.9	6, 10, 21	9a/b, 21, 22
9a	2.27 m	30.0	7, 8	8, 22 (w)
9b	2.65 m			8, 10
10	2.46 m	43.1		6, 9b, 14
11		44.9		
12a	1.38 m	44.4	10, 22	1a
12b	1.44 m		10, 22	22
13a	1.22 m	27.1	14, 15	22
13b	1.57 m			14, 23
14	1.87 m	51.4		10, 13b, 16, 23
15	2.61 m	31.9		17, 23
16	5.25 t (9.3)	136.0	14, 18	14, 17, 23
17	6.11 m	123.3	15, 19	16, 24
18	6.09 m	120.1	24, 25	15, 23, 25
19		135.3		
20	1.26 s	25.2	2, 3, 4	1b, 2, 4b
21	9.12 s	194.7	6, 7, 8	8
22	0.77 s	22.8	1, 10, 11	1b, 2, 8, 9a, 12b, 13a
23	0.92 d (6.6)	21.1	14, 15, 16	13b, 14, 15, 16, 17
24	1.70 s	17.9	18, 19, 25	17
25	1.79 s	26.1	18, 19, 24	17

7.8 Ophiobolin C isolated from *A. calidoustus* (IBT 25726)

Figure S14. ^{1}H-NMR spectrum of ophiobolin C in $CDCl_3$ at 800 MHz.

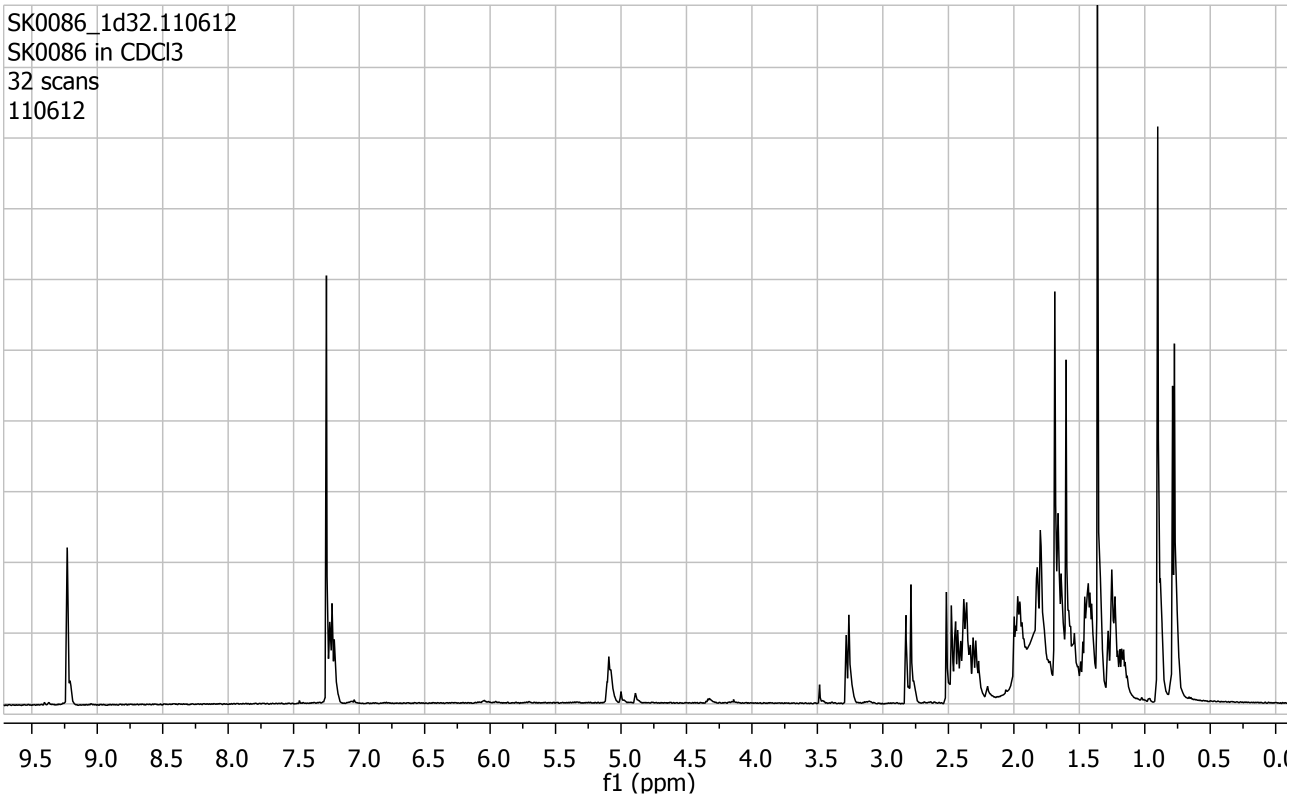

Table S15. ^{1}H and ^{13}C-NMR for ophiobolin C in $CDCl_3$ at 800 MHz for ^{1}H and 200 MHz for ^{13}C.

	δ_H mult. (*J* (Hz))	δ_C *	HMBC connectivities	NOE connectivities
1a	1.26 m	36.0	2, 10, 11	
1b	1.81 m		6, 10	2, 12a, 20
2	2.38 m	50.8		1b, 4a, 6, 20, 22
3		76.6		
4a	2.49 m	54.6	5, 20	2, 20
4b	2.80 m		2, 3, 5	20
5		217.5		
6	3.26 m	48.4	2, 3, 5, 7, 8, 21	2, 9a, 22
7		141.5		
8	7.21 m	163.8	6, 9, 21	9b, 10, 21
9a	2.31 m	24.7	7, 8, 10, 11	6, 22, 23
9b	2.45 m		7, 8, 10, 11	8, 10
10	1.67 m	53.4	8, 14, 15	8, 9b, 16b
11		43.6		
12a	1.41 m	42.6		1b, 22
12b	1.44 m			
13a	1.46 m	22.8	11	23
13b	1.55 m		10, 11	14
14	2.36 m	45.2		13b, 15, 16b, 17b
15	1.65 m	32.7	16	14, 23
16a	1.18 m	36.8	14, 15, 17, 18, 23	17a, 23
16b	1.24 m		14, 15, 17, 18, 23	1b, 10, 14,17b, 18
17a	1.95 m	26.0	15, 16, 18, 19	16a,18
17b	2.00 m		16, 18, 19	14, 16b, 23, 24
18	5.09 m	124.3	24, 25	16b, 17a, 25
19		131.0		
20	1.36 s	25.4	2, 3, 4	1b, 2, 4a/b
21	9.23 s	195.9	6, 7	8
22	0.90 s	19.0	10, 11, 12	2, 6, 9a, 12a
23	0.78 d (6.8)	16.4	14, 15, 16	9a, 13a, 15, 16a, 17b
24	1.61 s	17.6	18, 19, 25	17b
25	1.69 s	25.6	18, 19, 24	18

* ^{13}C NMR chemichal shifts determinated from HSQC and HMBC experiments.

7.9 6-epiophiobolin G isolated from *A. calidoustus* (IBT 25726)

Figure S15. ^{1}H-NMR spectrum of 6-epiophiobolin G in $CDCl_3$ at 800 MHz.

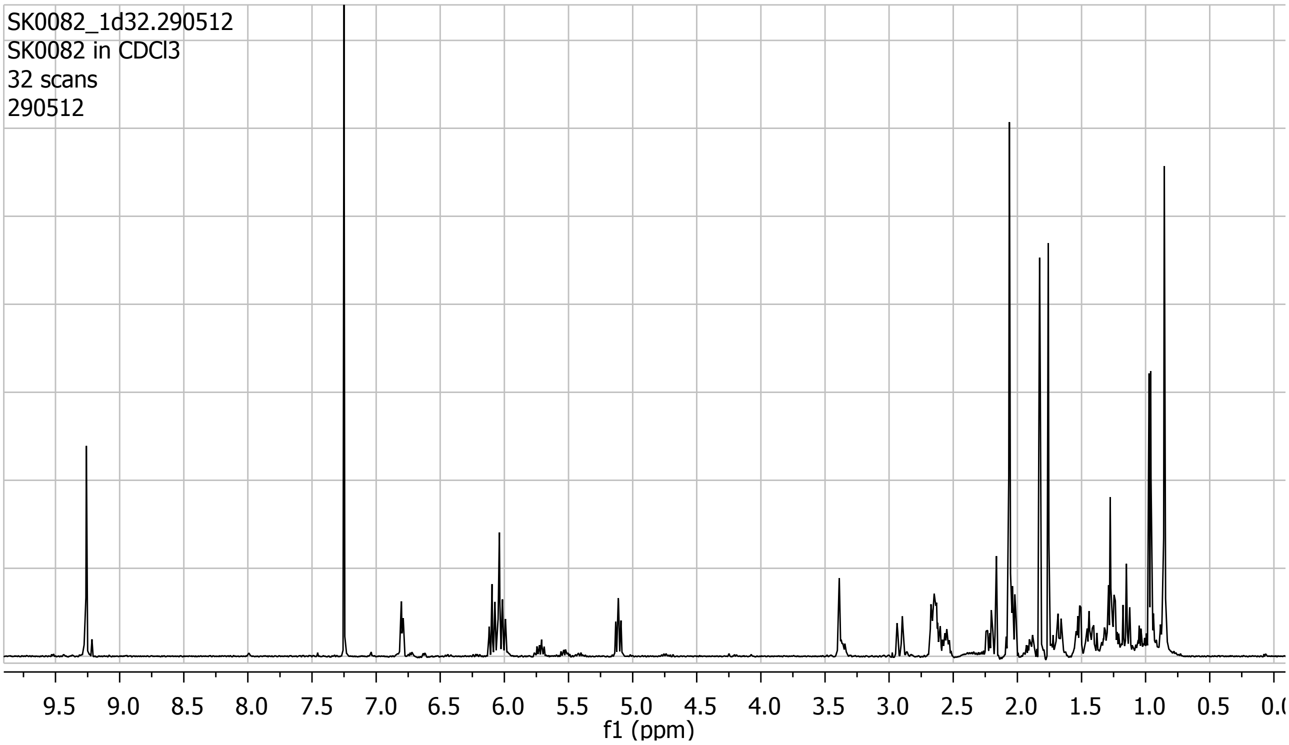

Figure S16. ^{13}C-NMR spectrum of 6-epiophiobolin G in $CDCl_3$ at 200 MHz.

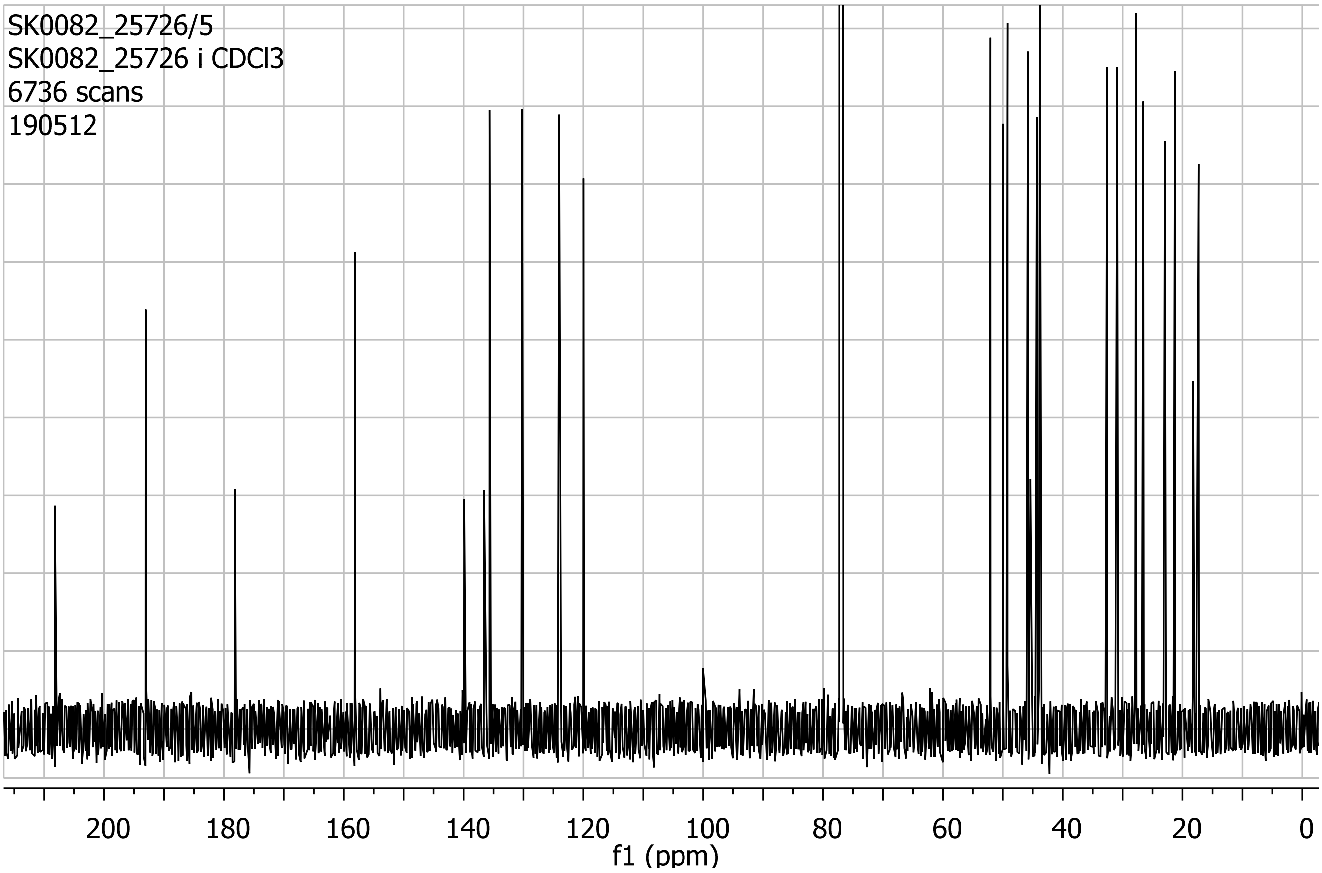

Table S16. ^{1}H and ^{13}C-NMR for 6-epiophiobolin G in $CDCl_3$ at 800 MHz for ^{1}H and 200 MHz for ^{13}C.

	δ_H mult. (J (Hz))	δ_C	HMBC connectivities	NOE connectivities
1a	1.15 m	45.8	2, 3, 10, 11, 22	6, 10, 12a
1b	2.03 m		3, 6, 12, 22	2, 22
2	2.66 m	49.2	1	1b, 22
3		178.1		
4	6.04 s	130.2	3, 5, 6, 20	20
5		208.2		
6	3.40 m	50.0	1, 2, 5, 7, 8, 21	1a, 10
7		139.9		
8	6.80 m	158.1	6, 9, 10, 21	9a/b, 21, 22
9a	2.20 m	30.9	7, 8, 10, 14	8, 15, 22
9b	2.93 m		7, 8, 10, 11	8, 10
10	2.63 m	43.8	8, 9, 11, 13, 14, 22	1a, 6, 9b, 14
11		45.4		
12a	1.43 m	44.3	1, 13, 14, 22	1a, 13b
12b	1.52 m		1, 10, 13, 14, 22	22
13a	1.25 m	27.8	12, 14, 15	22
13b	1.67 m		10, 11, 14	12a, 14, 23
14	1.89 m	52.1	9, 10, 13, 15, 16, 23	10, 13b, 16, 23
15	2.55 m	32.6	14, 16, 17, 23	9a/b, 18, 23
16	5.11 t (1.3)	135.7	14, 15, 17, 18, 23	14, 17, 23
17	6.10 m	124.0	15, 18, 19, 23	16, 24
18	6.00 m	120.0	16, 17, 24, 25	15, 23, 25
19		136.6		
20	2.06 s	17.3	2, 3, 4, 5	4
21	9.26 s	193.0	6, 7	8
22	0.85 s	22.9	1, 12	1b, 2, 8, 9a, 12b, 13a
23	0.97 d (6.8)	21.3	14, 15, 16	13b, 14, 15, 16,18
24	1.76 s	18.2	18,19, 25	17
25	1.83 s	26.5	18,19, 24	18

7.10 Activity of ophiobolin A, B, C and K + 6-epiophiobolin K towards healthy fibroblasts (Wi-38) cells

Figure S17. Activity of ophiobolin A, B, C and K + 6-epiophioboln K towards healthy fibroblasts (Wi-38).

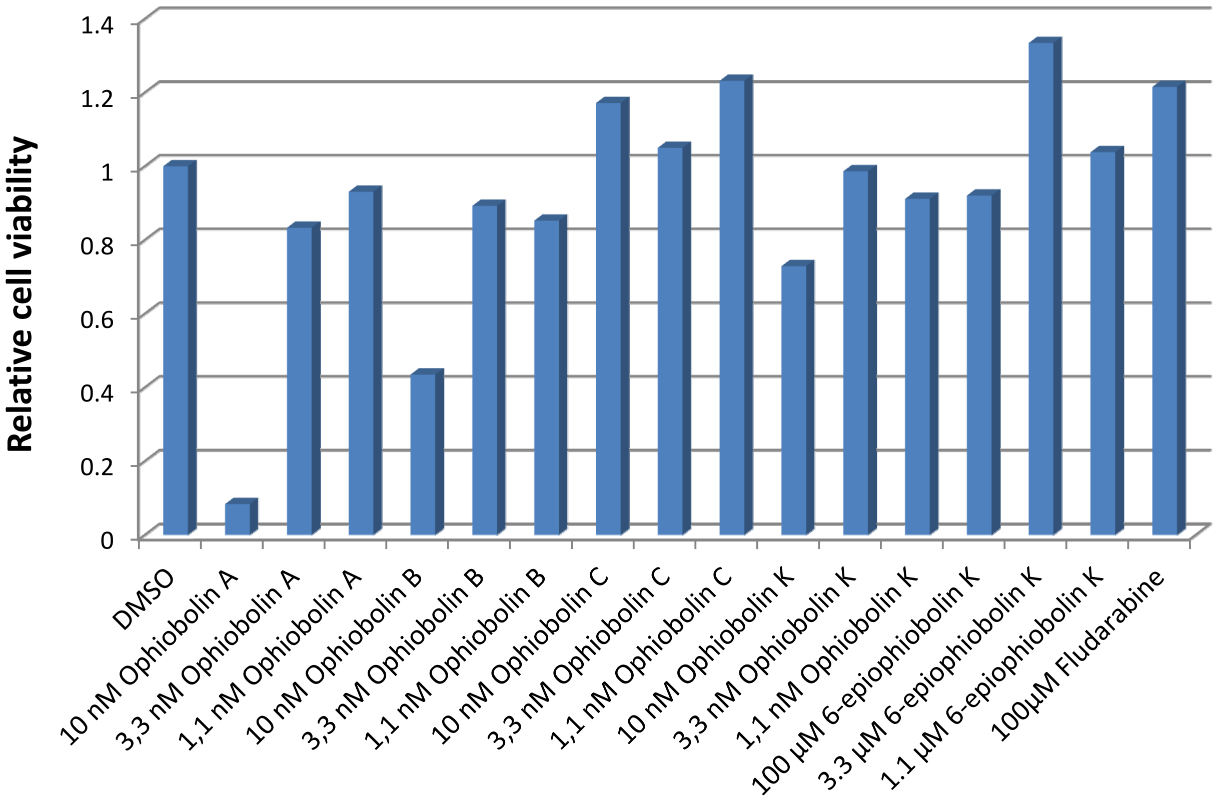

7.11 Apoptosis induction in CLL cells by different ophiobolins

Figure S18. CLL cells cultured in DMEM were treated for 24 hours with two different concentrations of ophiobolin A, ophiobolin B, ophiobolin C and ophiobolin K. In order to analyze apoptosis induction, cells of each well were divided into two parts and analyzed by flow cytometry using two different staining strategies. (**a**) cell survival was analyzed by gating on cells that were negative for Annexin V-phycoerythrin (PE) and 7-amino-actinomycin (7-AAD). Relative survival compared to DMSO control (0.1%) is depicted as mean values +SD of 4 independent CLL samples; (**b**) Caspase-3 activity is depicted as mean values +SD of 4 independent CLL samples relative to DMSO control (0.1%).

(**a**)

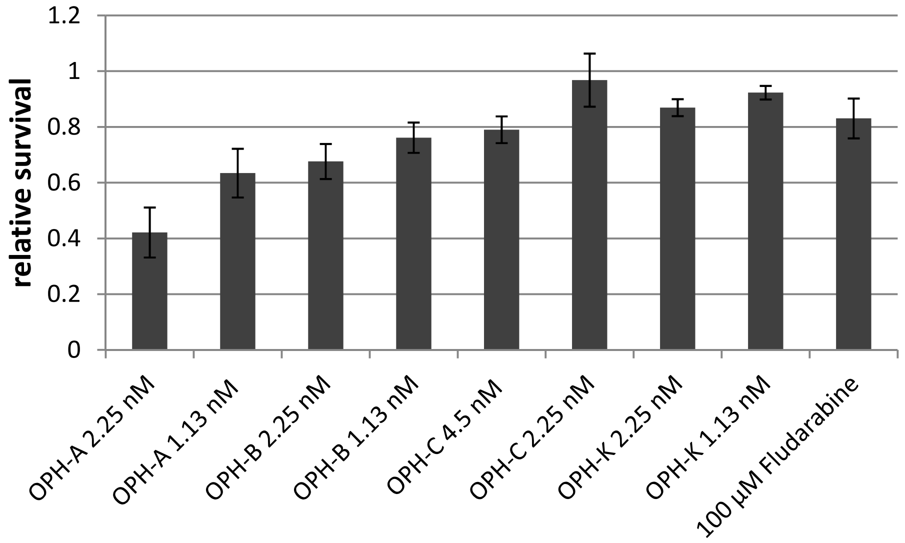

(**b**)

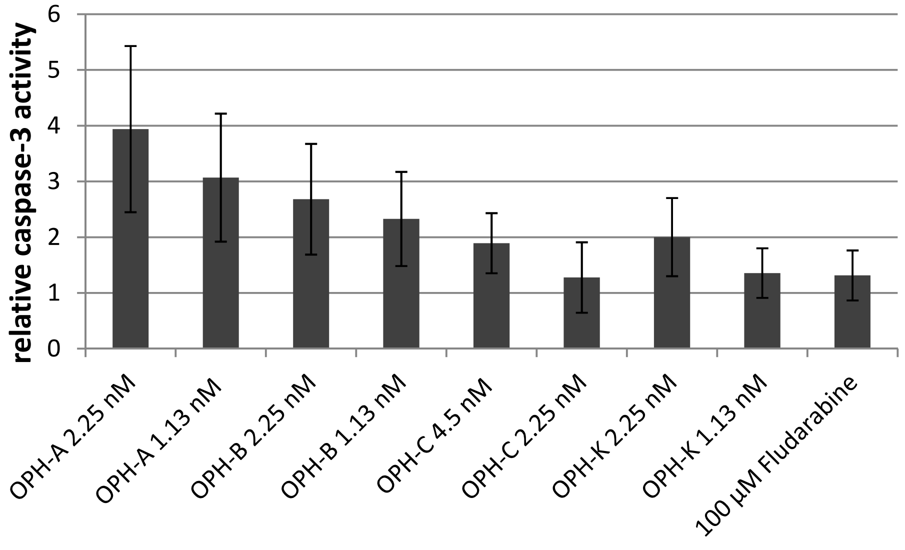

Supplement: Supplementary file 1 [file molecules-18-14629-s001.pdf]
